# Long-term effects of urban biophilic art environments on depression and anxiety symptoms: a longitudinal intervention study

**DOI:** 10.1186/s40359-026-04262-6

**Published:** 2026-03-04

**Authors:** Yu Hou, Qirui Zhang

**Affiliations:** 1 College of Design, Hanyang University, Seoul, South Korea; 2 College of Art, Anhui Xinhua University, Hefei, Anhui 230088 China

**Keywords:** Biophilic design, Urban mental health, Depression, Anxiety, Environmental psychology, Longitudinal intervention

## Abstract

Urban environments have been increasingly associated with elevated rates of depression and anxiety disorders, prompting exploration of innovative environmental interventions to promote mental health. This longitudinal study investigated the therapeutic effects of urban biophilic art environments on depression and anxiety symptoms among 300 urban residents over a 12-month period. Using a quasi-experimental design, participants were divided into intervention (*n* = 156) and control (*n* = 144) groups, with the intervention group having regular access to purpose-designed biophilic art installations integrating natural elements with artistic expression. Mental health outcomes were assessed using standardized measures (PHQ-9, GAD-7) at baseline, 3, 6, 9, and 12 months. Results demonstrated significant improvements in both depression (F(4,1192) = 18.47, *p* < .001, partial η² = 0.058) and anxiety symptoms (F(4,1192) = 17.15, *p* < .001, partial η² = 0.055) in the intervention group compared to controls. Mediation analysis revealed that attention restoration, stress reduction, and environmental perception quality accounted for 67% of depression symptom improvement and 72% of anxiety symptom reduction. Individual differences in nature connectedness and age moderated intervention effectiveness. These findings provide evidence that biophilic art environments can serve as accessible, community-based interventions for urban mental health promotion, offering practical implications for urban planning and public health policy.

**Supplementary Information:**

The online version contains supplementary material available at 10.1186/s40359-026-04262-6.

## Introduction

The rapid pace of urbanization has fundamentally transformed human living environments, with over half of the global population now residing in urban areas, a figure projected to reach 68% by 2050 [1]. This unprecedented urban expansion has coincided with a significant deterioration in mental health outcomes, particularly evident in the rising prevalence of depression and anxiety disorders among urban populations [2]. Contemporary urban environments, characterized by dense built infrastructure, limited natural elements, and high-stress living conditions, have been increasingly recognized as contributing factors to the growing mental health crisis affecting millions of individuals worldwide [3].

In response to these mounting concerns, environmental design disciplines have begun exploring innovative approaches to integrate natural elements into urban settings, giving rise to the concept of biophilic design [4]. Biophilic art environments represent a particularly promising evolution of this design philosophy, combining the psychological benefits of nature connection with the emotional and aesthetic impact of artistic expression [5]. These environments seek to address the fundamental human need for nature contact while simultaneously providing culturally enriching experiences that can enhance psychological well-being and community engagement.

The theoretical foundation for biophilic art environments draws upon extensive research demonstrating the restorative effects of natural environments on human psychology and physiology [6]. However, the specific mechanisms through which long-term exposure to artistically enhanced biophilic environments might influence mental health outcomes, particularly in relation to depression and anxiety symptoms, remain inadequately understood. While previous studies have established the short-term benefits of nature exposure and art engagement on mood and stress reduction, the cumulative effects of sustained interaction with integrated biophilic art environments have not been systematically investigated [7]. Recent meta-analyses and systematic reviews have further confirmed that nature-based interventions yield moderate to large effect sizes for reducing depression and anxiety symptoms [8], yet the potential of combining biophilic elements with artistic expression in urban contexts remains largely unexplored.

This research addresses a critical gap in the current literature by examining the long-term psychological impacts of biophilic art environments on urban residents’ mental health. The study specifically focuses on the potential of these environments to serve as accessible, community-based interventions for depression and anxiety symptom management, representing a novel approach to urban mental health promotion that bridges environmental psychology, therapeutic design, and public art initiatives [9].

The primary objective of this research is to investigate the relationship between long-term exposure to urban biophilic art environments and the alleviation of depression and anxiety symptoms among urban residents. Specifically, this study seeks to address the following research questions: First, how does sustained exposure to biophilic art environments influence the severity and frequency of depressive symptoms in urban populations? Second, what are the mechanisms through which these environments contribute to anxiety reduction and emotional regulation? Third, what design characteristics of biophilic art environments are most effective in promoting mental health benefits? Finally, how do individual differences in personality, demographic factors, and baseline mental health status moderate the relationship between environmental exposure and psychological outcomes?

This research makes several significant contributions to the fields of environmental psychology, urban design, and public health. From a theoretical perspective, the study extends existing knowledge about human-environment interactions by examining the synergistic effects of natural elements and artistic interventions on mental health outcomes. Unlike previous short-term studies, our 12-month longitudinal design captures cumulative exposure effects that may require extended periods to manifest fully. The findings contribute to the development of evidence-based design principles for creating therapeutic urban environments that can serve both aesthetic and psychological functions. From a practical standpoint, this research provides crucial insights for urban planners, public health officials, and community developers seeking cost-effective, accessible interventions for addressing the mental health challenges associated with urban living. The identification of specific design elements and exposure patterns that maximize therapeutic benefits can inform future urban development projects and public art initiatives, potentially creating more mentally healthy cities for millions of residents.

This paper is structured to provide a comprehensive examination of biophilic art environments and their impact on mental health outcomes. Following this introduction, the literature review synthesizes existing research on biophilic design, environmental psychology, and art therapy to establish the theoretical framework for the study. The methodology section details the longitudinal research design, participant recruitment procedures, measurement instruments, and analytical approaches employed to investigate the research questions. The results section presents findings related to the relationship between environmental exposure and mental health outcomes, including analyses of moderating factors and dose-response relationships. The discussion interprets these findings within the broader context of urban health promotion and environmental design, while the conclusion summarizes key contributions and outlines directions for future research and practical implementation.

Through this systematic investigation, the paper aims to advance understanding of how thoughtfully designed urban environments can serve as powerful tools for promoting mental health and well-being, ultimately contributing to the development of more psychologically supportive cities that enhance the quality of life for urban populations worldwide.

## Literature review and theoretical foundation

### Biophilic theory and urban art environments

The biophilic hypothesis, first articulated by Edward O. Wilson in the 1980s, posits that humans possess an innate affinity for life and life-like processes, representing an evolutionary adaptation that has shaped human psychological and physiological responses to natural environments [10]. This foundational theory has evolved from its initial biological origins to encompass a comprehensive framework for understanding human-nature relationships in contemporary built environments, particularly gaining prominence in architectural and urban design disciplines over the past three decades [11]. The core tenets of biophilic theory emphasize that regular contact with natural elements is not merely beneficial but essential for optimal human functioning, cognitive performance, and emotional well-being, establishing a scientific basis for incorporating nature-inspired design elements into urban spaces [12].

Contemporary applications of biophilic theory in urban art environments manifest through various design strategies that integrate natural forms, processes, and experiences with artistic expression. These manifestations include the incorporation of living vegetation as sculptural elements, the use of natural materials and textures in public art installations, and the creation of water features that combine aesthetic appeal with the restorative sounds and movements of natural water systems [13]. Additionally, biophilic art environments frequently employ biomimetic design principles, where artistic forms replicate natural patterns, structures, and processes, creating visual and spatial experiences that trigger positive psychological responses associated with nature exposure even in highly urbanized settings.

The design principles governing effective biophilic art environments can be mathematically conceptualized through the Biophilic Environmental Effectiveness Index (BEEI), which quantifies the psychological impact of natural elements within artistic contexts:

$$\boldsymbol B\boldsymbol E\boldsymbol E\boldsymbol I\boldsymbol=\mathbf{\left(\textstyle\sum\left(Ni\times Ai\right)\right)}\boldsymbol/\mathbf{\left({T\times D}\right)}$$

Where Ni represents the number of biophilic elements of type i, Wi indicates the psychological weight factor for element type i, Ai denotes the accessibility score for element i, T represents total exposure time, and D indicates the distance factor from the observer [14].

The implementation pathways for creating successful biophilic art environments involve several critical considerations that bridge ecological science, environmental psychology, and artistic practice. Primary among these is the strategic selection and placement of natural elements that maximize both aesthetic impact and psychological benefit, requiring careful attention to factors such as seasonal variability, maintenance requirements, and community accessibility [15]. Effective implementation also necessitates collaboration between artists, environmental psychologists, horticulturists, and urban planners to ensure that biophilic art installations not only achieve their intended aesthetic goals but also deliver measurable improvements in mental health outcomes for urban populations.

The theoretical framework supporting biophilic art environments extends beyond simple nature incorporation to encompass complex interactions between sensory experience, cultural meaning, and psychological restoration. These environments function as hybrid spaces that simultaneously serve aesthetic, ecological, and therapeutic functions, creating opportunities for urban residents to experience the psychological benefits of nature contact within culturally enriching contexts that enhance community engagement and social cohesion.

### Psychological health mechanisms from environmental psychology perspective

Environmental psychology provides a comprehensive theoretical framework for understanding how physical environments influence human psychological functioning, with particular emphasis on the mechanisms through which natural and nature-inspired environments promote mental health and emotional well-being. The field has identified several key pathways through which environmental features interact with cognitive and emotional processes, establishing empirical foundations for the therapeutic potential of biophilic art environments in urban settings [16]. These mechanisms operate through complex interactions between sensory perception, cognitive processing, and physiological responses, creating measurable improvements in psychological outcomes through sustained environmental exposure. Recent advances in neuroimaging and psychophysiological assessment have provided deeper insights into these underlying processes [17].

Attention Restoration Theory (ART), developed by Kaplan and Kaplan, represents one of the most influential frameworks for understanding the psychological benefits of natural environments. According to ART, natural settings possess four key characteristics that promote cognitive restoration: being away from routine mental demands, fascination with environmental features, extent or scope of the environment, and compatibility between individual inclinations and environmental affordances [18]. In biophilic art environments, these restorative characteristics manifest through carefully designed features that capture involuntary attention while reducing the cognitive load associated with urban stress, thereby allowing directed attention mechanisms to recover from mental fatigue and enhancing overall psychological resilience.

The connection between ART and depression symptom alleviation operates through several interrelated mechanisms. Depression is frequently characterized by cognitive deficits including impaired concentration, difficulty sustaining attention, and mental fatigue [19]. By restoring directed attention capacity, biophilic environments help reduce the cognitive burden that often exacerbates depressive rumination. When individuals experience “soft fascination” in natural settings, the mind shifts away from repetitive negative thought patterns toward effortless engagement with pleasant environmental stimuli. Research has demonstrated that nature walks can reduce activity in the subgenual prefrontal cortex, a brain region associated with repetitive negative thinking, thereby interrupting the rumination cycles that maintain depressive states [20]. Additionally, the restoration of attention resources alleviates feelings of mental exhaustion and burnout, leading to positive changes in mood and self-efficacy that counteract depressive symptoms [21].

Similarly, ART explains anxiety reduction through cognitive restoration pathways. Anxiety disorders involve hypervigilance, excessive worry, and difficulty disengaging from threat-related stimuli, all of which deplete directed attention resources. Natural environments provide a sanctuary where the effortful attention required for threat monitoring can be suspended. The “being away” component allows psychological distance from anxiety-provoking concerns, while “fascination” redirects attention toward calming natural stimuli. Research indicates that even brief exposure to natural environments or nature images can improve attentional performance and reduce anxiety-related cognitive biases [22]. The compatible and extensive qualities of well-designed biophilic environments further promote a sense of safety and coherence that counters the unpredictability and threat perception underlying anxiety disorders.

Stress Reduction Theory (SRT), alternatively known as Psycho-evolutionary Stress Recovery Theory, posits that exposure to natural environments triggers immediate physiological and psychological responses that counteract stress-related arousal patterns. This theory suggests that biophilic environments activate the parasympathetic nervous system, leading to reduced cortisol levels, decreased heart rate, and lowered blood pressure, while simultaneously promoting positive emotional states and reducing anxiety symptoms [23]. The artistic integration of natural elements in urban environments amplifies these stress-reduction effects by creating aesthetically pleasing experiences that engage multiple sensory modalities and promote deeper psychological engagement with restorative environmental features.

The pathway from SRT to depression mitigation involves both direct physiological and indirect psychological mechanisms. Chronic stress and elevated cortisol levels are strongly implicated in the development and maintenance of depression, disrupting neurotransmitter systems and promoting inflammation that affects mood regulation [24]. By reducing physiological stress markers, biophilic environments may help normalize the biological dysregulation underlying depressive symptoms. Furthermore, the positive affective responses elicited by natural and aesthetically pleasing environments directly counteract the anhedonia (inability to experience pleasure) characteristic of depression. The multi-sensory engagement provided by biophilic art environments, including visual beauty, natural sounds, and tactile experiences with natural materials, stimulates reward pathways and promotes positive emotional states.

For anxiety, SRT provides a direct mechanism of symptom reduction. Anxiety is fundamentally linked to sympathetic nervous system activation and the stress response. Natural environments rapidly down-regulate this arousal, often within minutes of exposure [23]. The parasympathetic activation induced by nature contact promotes a relaxation response that is incompatible with anxiety. Heart rate variability increases, muscle tension decreases, and physiological markers of stress normalize. These physiological changes create a bodily state that signals safety to the brain, reducing subjective anxiety and promoting emotional equilibrium. In biophilic art environments, the combination of natural elements with artistic expression may enhance these effects by providing multiple channels of positive stimulation that reinforce the calming response.

Emotion Regulation Theory provides a third critical perspective on the psychological mechanisms underlying biophilic environment benefits, focusing on how environmental factors influence emotional processing and affective states. Natural and nature-inspired environments facilitate emotion regulation through multiple pathways, including the enhancement of positive affect, the reduction of rumination patterns associated with depression and anxiety, and the promotion of mindfulness and present-moment awareness [25]. A recent systematic review confirmed that nature-based interventions effectively reduce stress, anxiety, and depression levels through these emotion regulation mechanisms [26]. The mathematical relationship between environmental exposure and emotional regulation can be expressed through the Environmental Emotion Regulation Index (EERI):

$$\boldsymbol E\boldsymbol E\boldsymbol R\boldsymbol I\boldsymbol=\mathbf{\left({\triangle PA\times\alpha t+\triangle NA\times\beta t}\right)}\boldsymbol/\mathbf{\left({Et\times It}\right)}$$

Where ΔPA represents change in positive affect, ΔNA indicates change in negative affect, αt and βt are time-dependent weighting coefficients, Et denotes environmental exposure intensity, and It represents individual susceptibility factors [27].

The integration of these theoretical perspectives reveals that biophilic art environments operate through multiple complementary mechanisms to promote psychological health. The cognitive restoration provided by ART combines with the physiological stress reduction described by Stress Relief Theory and the emotional benefits outlined in Emotion Regulation Theory to create synergistic effects that exceed the sum of individual components [28]. Recent research has emphasized the importance of viewing these benefits through an ecosystem services lens, integrating mental health outcomes into broader frameworks of human-nature interaction [29]. This multi-pathway approach explains why biophilic art environments demonstrate particular effectiveness in addressing complex mental health challenges such as depression and anxiety, which involve cognitive, physiological, and emotional dimensions that can be simultaneously addressed through comprehensive environmental interventions that engage multiple psychological systems.

### Environmental intervention research on depression and anxiety symptoms

Empirical research investigating environmental interventions for depression and anxiety symptoms has gained considerable momentum over the past two decades, with studies consistently demonstrating the therapeutic potential of nature-based and environmentally enhanced interventions across diverse populations and settings. Longitudinal studies conducted in various cultural contexts have provided compelling evidence that structured exposure to natural environments can significantly reduce depressive symptom severity, with effect sizes ranging from moderate to large depending on intervention duration, environmental characteristics, and participant demographics [20]. These findings have established a robust foundation for understanding how environmental modifications can serve as adjunctive or standalone treatments for mood disorders, particularly in community-based settings where traditional therapeutic resources may be limited or inaccessible.

Research focusing specifically on depression interventions has revealed that green space exposure, horticultural therapy, and wilderness-based programs demonstrate measurable improvements in mood regulation, self-esteem, and overall psychological functioning. A comprehensive meta-analysis examining 47 studies found that participants engaging in nature-based interventions showed significant reductions in depressive symptoms compared to control groups, with particular effectiveness observed in programs lasting eight weeks or longer [30]. More recent meta-analytic evidence has confirmed these findings, with one 2024 study reporting that nature exposure yields moderately sized effects on adults with mental illness symptoms, supporting the integration of nature-based approaches into treatment planning [31]. The therapeutic mechanisms underlying these improvements appear to involve both direct physiological effects, such as increased vitamin D synthesis and reduced cortisol production, and indirect psychological benefits, including enhanced social connection, increased physical activity, and improved sleep quality.

Anxiety-focused environmental intervention research has similarly demonstrated substantial therapeutic benefits, with studies indicating that exposure to natural environments can reduce both trait and state anxiety levels through multiple pathways. Forest bathing interventions, garden-based therapy programs, and urban green space utilization have all shown significant anxiolytic effects, with physiological measures such as reduced heart rate variability and decreased stress hormone levels providing objective evidence of intervention effectiveness [32]. A recent meta-analysis found that virtual nature exposure effectively reduces anxiety levels with a large effect size (SMD = 0.82), stress with a moderate effect size (SMD = 0.577), and depression with a moderate effect size (SMD = 0.621), suggesting that even simulated nature experiences can provide meaningful benefits [33]. The anxiolytic effects of environmental interventions appear to be mediated by enhanced parasympathetic nervous system activation and improved emotion regulation capacity, particularly in populations experiencing chronic stress or anxiety disorders.

The strengths of existing environmental intervention research include robust methodological designs, diverse participant populations, and consistent replication of findings across multiple cultural and geographic contexts. Many studies have employed randomized controlled trial designs with adequate sample sizes, standardized outcome measures, and appropriate follow-up periods to assess intervention durability [34]. A 2024 systematic review of biophilic design in healthcare settings found that such design approaches reduce hospitalization time, patient mortality, pain levels, and stress for healthcare providers while alleviating anxiety and supporting faster recovery [35]. Additionally, the integration of both subjective self-report measures and objective physiological indicators has strengthened the evidence base by providing convergent validity for intervention effects and illuminating the biological mechanisms underlying therapeutic benefits.

However, several limitations in the current literature create opportunities for advancing the field through more sophisticated research approaches. Most existing studies have focused on short-term interventions lasting weeks to months, with limited investigation of long-term exposure effects and optimal dosage parameters for sustained therapeutic benefits [36]. Furthermore, the majority of research has examined traditional natural environments rather than hybrid spaces that combine natural elements with artistic or cultural features, leaving significant gaps in understanding how biophilic art environments might enhance or modify therapeutic outcomes. Systematic reviews have also noted methodological variations across studies and the need for standardized approaches to measuring nature exposure and mental health outcomes [26].

The Environmental Intervention Effectiveness Coefficient (EIEC) can quantify the relationship between intervention characteristics and therapeutic outcomes:

$$\boldsymbol E\boldsymbol I\boldsymbol E\boldsymbol C\boldsymbol=\mathbf{\left(\textstyle\sum\left(Ii\times Di\times Fi\right)\right)}\boldsymbol/\mathbf{\left({Bi\times Ti\times Ri}\right)}$$

Where Ii represents intervention intensity, Di indicates duration of exposure, Fi denotes frequency of contact, Bi represents baseline symptom severity, Ti indicates time since intervention initiation, and Ri represents individual resilience factors [37].

The identified research gaps provide clear justification for investigating long-term exposure to biophilic art environments as a novel therapeutic approach that addresses limitations in existing literature while building upon established theoretical foundations and empirical evidence supporting environmental interventions for mental health promotion.

## Research design and methods

### Research framework and hypothesis construction

The theoretical model underlying this research integrates biophilic theory, environmental psychology principles, and mental health intervention frameworks to establish a comprehensive understanding of how urban biophilic art environments influence psychological outcomes through sustained exposure. The conceptual framework posits that biophilic art environments function as therapeutic interventions through multiple interconnected pathways, including direct physiological effects, cognitive restoration processes, and emotional regulation mechanisms that collectively contribute to reduced depression and anxiety symptoms [38].

**Fig. 1:**
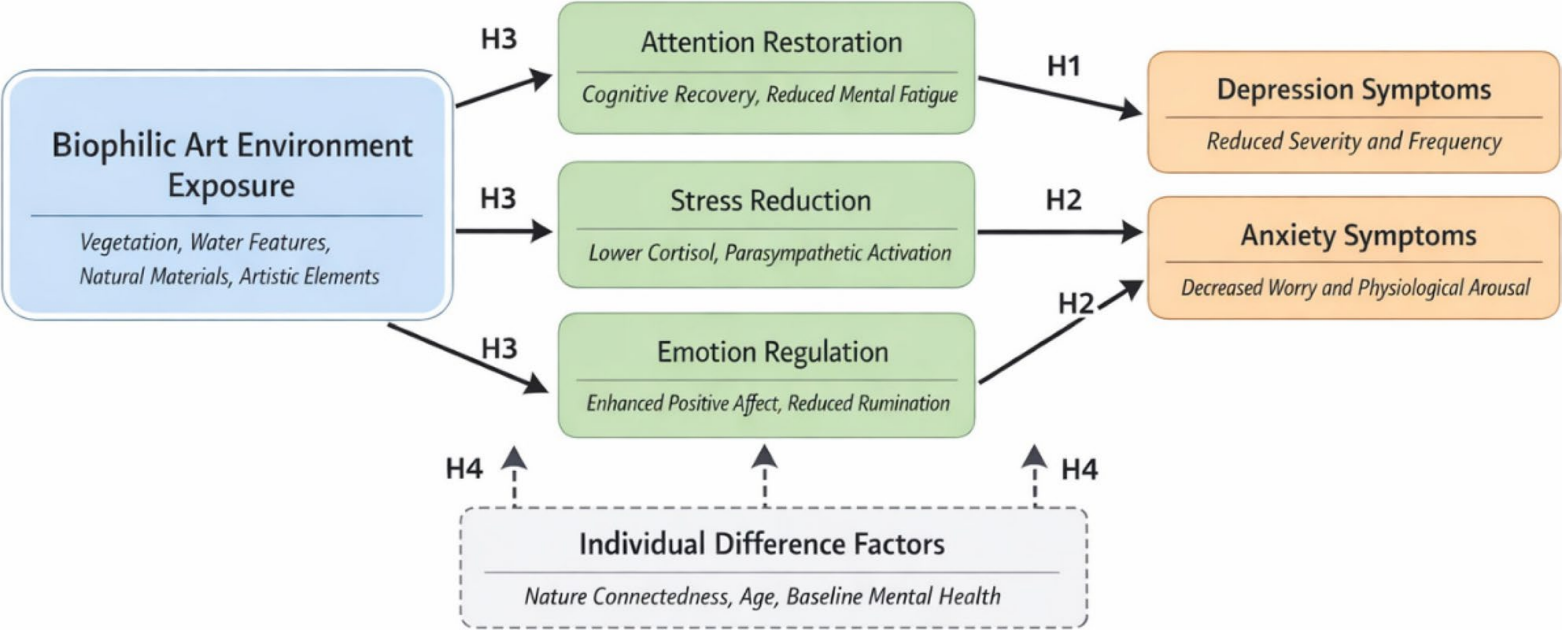
Proposed theoretical model of urban biophilic art environment effects on mental health. Note: This figure depicts the hypothesized conceptual framework based on Attention Restoration Theory, Stress Reduction Theory, and Emotion Regulation Theory. Arrows indicate proposed causal pathways. H1-H4 denote the four research hypotheses to be tested empirically. The empirical path coefficients from structural equation modeling analysis are presented separately in Fig. 5 within the Results section

As illustrated in Fig. 1, the proposed theoretical model demonstrates the hypothesized relationships between environmental exposure variables, mediating psychological processes, and mental health outcomes, while accounting for individual difference factors that may moderate these relationships. It is important to note that this figure represents a conceptual framework derived from existing theory rather than empirical results; the path coefficients shown represent hypothesized directions of influence to be tested in this study.

Based on this theoretical foundation, the research proposes four primary hypotheses that will be tested through longitudinal investigation. Hypothesis 1 predicts that individuals with greater long-term exposure to urban biophilic art environments will demonstrate significantly lower levels of depressive symptoms compared to those with minimal exposure, with this relationship strengthening over time as cumulative exposure effects accumulate [39]. Hypothesis 2 posits that sustained contact with biophilic art environments will result in measurable reductions in anxiety symptom severity, with particular effectiveness observed for individuals experiencing moderate to high baseline anxiety levels. Hypothesis 3 suggests that the relationship between environmental exposure and mental health outcomes will be mediated by improvements in attention capacity, stress resilience, and emotional regulation skills, as predicted by environmental psychology theories. Hypothesis 4 proposes that individual difference factors, including personality traits, demographic characteristics, and baseline mental health status, will moderate the strength and direction of relationships between environmental exposure and psychological outcomes.

The causal pathway model incorporates both direct and indirect effects of biophilic art environment exposure on mental health outcomes. The direct pathway model can be expressed mathematically as:

$$\boldsymbol M\boldsymbol H\boldsymbol={\mathbf\beta}_{\mathbf0}\boldsymbol+{\mathbf\beta}_{\mathbf1}\mathbf{\left({BAE}\right)}\boldsymbol+{\mathbf\beta}_{\mathbf2}\mathbf{\left(T\right)}\boldsymbol+{\mathbf\beta}_{\mathbf3}\mathbf{\left({BAE\times T}\right)}\boldsymbol+\mathbf\epsilon$$

Where MH represents mental health outcomes, BAE indicates biophilic art environment exposure intensity, T denotes time, and ε represents error variance [40].

The comprehensive research framework requires careful specification of control variables and moderating factors that may influence the primary relationships under investigation. As shown in Table 1, the operationalization of key research variables provides clear definitions and measurement approaches for each construct included in the theoretical model. Control variables include demographic factors such as age, gender, education level, and socioeconomic status, as well as baseline mental health indicators and concurrent life stressors that might confound the relationship between environmental exposure and psychological outcomes [41]. Moderating variables encompass personality traits such as nature connectedness, openness to experience, and environmental sensitivity, which may amplify or diminish the therapeutic effects of biophilic art environment exposure.

**Table 1:** Operationalization of research variables

Variable Name	Variable Type	Conceptual Definition	Operational Definition	Measurement Method
Biophilic Art Environment Exposure	Independent Variable	The degree of contact between individuals and environments that integrate natural elements with artistic expression, encompassing physical presence, sensory engagement, and temporal duration	Frequency and duration of contact with designated biophilic art installations over 12-month period	GPS tracking, self-report logs, observational data
Depression Symptoms	Dependent Variable	A persistent affective state characterized by low mood, loss of interest or pleasure, and associated cognitive-behavioral symptoms as defined by DSM-5 diagnostic criteria	Severity of depressive symptomatology assessed using standardized clinical measures	Patient Health Questionnaire-9 (PHQ-9)
Anxiety Symptoms	Dependent Variable	A psychological state characterized by excessive worry, apprehension, and physiological arousal that interferes with daily functioning	Level of anxiety symptoms and associated functional impairment	Generalized Anxiety Disorder-7 (GAD-7)
Attention Restoration	Mediating Variable	The recovery of depleted directed attention capacity following exposure to environments that engage involuntary attention through soft fascination	Cognitive capacity recovery following directed attention fatigue	Attention Restoration Scale (ARS)
Stress Levels	Mediating Variable	The magnitude of physiological and psychological arousal resulting from perceived demands exceeding adaptive capacity	Physiological and psychological stress indicators	Perceived Stress Scale, cortisol measurements
Nature Connectedness	Moderating Variable	An individual’s subjective sense of psychological relationship, identification, and emotional bond with the natural world	Individual’s psychological connection and identification with natural environments	Nature Relatedness Scale (NR-6)
Age	Control Variable	The chronological duration of life from birth	Chronological age of participants	Demographic questionnaire
Baseline Mental Health	Control Variable	Pre-existing psychological functioning status prior to intervention exposure	Pre-intervention psychological functioning levels	Comprehensive mental health assessment battery

The mediated moderation model examining the interaction between exposure characteristics and individual difference factors can be represented as:

$$\begin{aligned} \mathbf Y\boldsymbol=&\:\mathbf\alpha\boldsymbol+{\mathbf c}_{\mathbf1}\mathbf X\boldsymbol+{\mathbf c}_{\mathbf2}\mathbf M\boldsymbol+{\mathbf c}_{\mathbf3}\mathbf W\boldsymbol+{\mathbf c}_{\mathbf4}\mathbf{\left({X\times W}\right)}\\&\boldsymbol +{\mathbf c}_{\mathbf5}\mathbf{\left({M\times W}\right)}\boldsymbol+{\mathbf c}_{\mathbf6}\mathbf{\left({X\times M\times W}\right)}\boldsymbol+\mathbf e \end{aligned}$$

Where Y represents mental health outcomes, X indicates environmental exposure, M denotes mediating variables, W represents moderating factors, and interaction terms capture the complex relationships between these variables.

This comprehensive research framework provides the theoretical foundation for empirically testing the proposed relationships while controlling for confounding factors and examining the boundary conditions under which biophilic art environments demonstrate optimal therapeutic effectiveness for depression and anxiety symptom reduction.

### Experimental design and data collection

This research employs a quasi-experimental longitudinal design to investigate the effects of sustained exposure to urban biophilic art environments on depression and anxiety symptoms over a 12-month period. The quasi-experimental approach was selected due to ethical considerations regarding random assignment to environmental conditions and the practical constraints of manipulating real-world urban environments, while still maintaining sufficient scientific rigor to establish causal inferences about environmental exposure effects [42]. The study design incorporates multiple measurement time points, matched comparison groups, and comprehensive control variable assessment to strengthen internal validity and minimize threats to causal interpretation.

Participant recruitment follows a stratified purposive sampling strategy targeting urban residents aged 18–65 years who meet specific inclusion criteria designed to ensure sample homogeneity while maintaining ecological validity. Inclusion criteria require participants to be permanent residents of the target urban area for at least two years, demonstrate stable housing situations, report mild to moderate depression or anxiety symptoms on screening measures, and have no current involvement in intensive mental health treatment programs [43]. Exclusion criteria eliminate individuals with severe mental health conditions requiring immediate clinical intervention, those with significant cognitive impairments that would affect data collection validity, and participants planning to relocate during the study period.

The experimental design consists of two primary groups: an intervention group with regular access to newly installed biophilic art environments and a matched comparison group residing in similar urban areas without access to these specialized environments. The intervention involves exposure to purpose-designed biophilic art installations that integrate living plants, natural materials, water features, and artistic elements following established biophilic design principles, with installations strategically placed in high-traffic community areas to maximize natural exposure opportunities [44].

The study was conducted in Seoul, South Korea, specifically within the Seongdong-gu and Gwangjin-gu districts, which share similar demographic profiles, population densities, and urban infrastructure characteristics. The intervention sites consisted of three biophilic art installations located in Seoul Forest Park (Seongdong-gu), along the Jungnangcheon Stream walking path (Gwangjin-gu), and within the Ttukseom Hangang Park area (Gwangjin-gu). These locations were selected based on accessibility, daily foot traffic patterns, and the feasibility of installing permanent biophilic art features. Control group participants resided in adjacent neighborhoods (Dongdaemun-gu and Jungnang-gu) with comparable urbanization levels but without access to the specialized biophilic art installations.

**Fig. 2:**
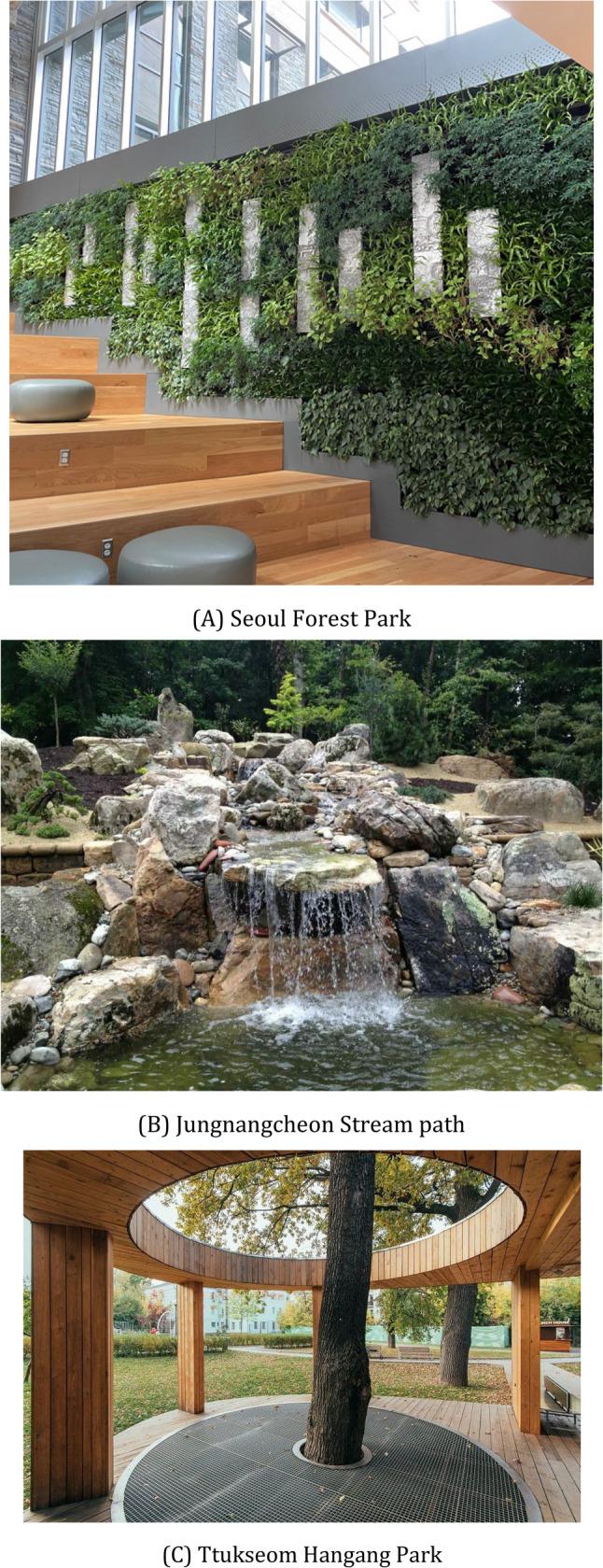
Biophilic art installation sites. **A** Seoul Forest Park, (**B**) Jungnangcheon Stream path, (**C**) Ttukseom Hangang Park. Panel **A** Seoul Forest Park installation featuring living green walls with integrated sculptural elements. Panel **B** Jungnangcheon Stream path installation incorporating water features and natural stone seating areas. Panel **C** Ttukseom Hangang Park installation with timber pavilion structures and native plantings. Photographs taken during the intervention period (May-September 2024)

The biophilic art installations incorporated multiple design elements derived from established biophilic design frameworks [45]. Each installation featured: (a) living green walls with native Korean plant species including Liriope platyphylla, Ophiopogon japonicus, and various fern varieties maintained through automated irrigation systems; (b) sculptural elements constructed from natural materials including locally sourced granite, untreated timber, and recycled wood; (c) water features incorporating gentle flowing water sounds and visual movement; and (d) artistic components including nature-inspired sculptures, mosaic patterns reflecting natural forms, and ambient lighting designed to complement natural daylight cycles. Fig. 2 presents representative photographs of the three intervention sites, illustrating the integration of biophilic and artistic elements.

The data collection procedure followed a standardized protocol implemented by a team of six trained research assistants who completed a 40-hour training program covering assessment administration, ethical conduct, and data quality assurance. Each assessment session lasted approximately 45–60 min and included the following sequence: informed consent verification, demographic questionnaire completion, administration of psychological measures (PHQ-9, GAD-7, Attention Restoration Scale, Perceived Stress Scale, Nature Relatedness Scale), physiological data collection, and debriefing.

Physiological assessment included salivary cortisol collection following established protocols. Participants were instructed to refrain from eating, drinking (except water), smoking, or brushing teeth for at least 30 min prior to sample collection. Saliva samples were collected using Salivette devices (Sarstedt, Germany) at two time points during each assessment session: upon arrival and 30 min post-arrival. Samples were immediately stored on ice and transported to the laboratory within 4 h, where they were centrifuged and frozen at -20 °C until analysis using enzyme-linked immunosorbent assay (ELISA) kits. Heart rate variability was measured using Polar H10 chest strap monitors during a standardized 5-minute seated rest period, with data analyzed for time-domain (RMSSD, SDNN) and frequency-domain (LF/HF ratio) parameters.

GPS tracking was implemented using a custom smartphone application developed for this study, which recorded participant location at 5-minute intervals during waking hours. Participants were instructed to carry their smartphones and keep location services activated throughout the study period. The application automatically detected when participants entered designated biophilic art environment zones (defined as 100-meter radius areas surrounding each installation) and logged duration of visits. Weekly self-report exposure logs complemented GPS data, allowing participants to record subjective engagement quality and activities during environmental visits.

**Fig. 3:**
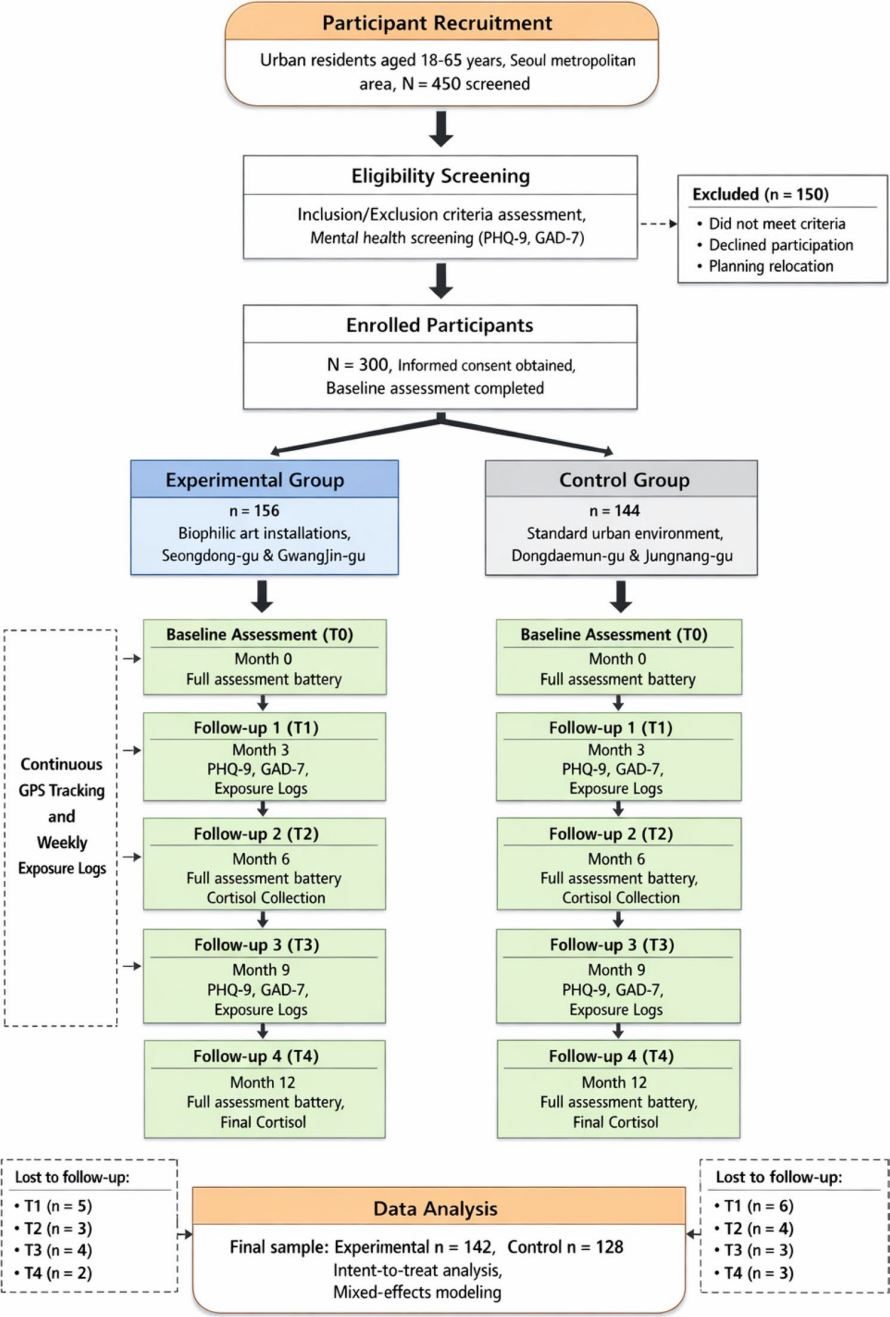
Experimental design flow chart and data collection procedure. Note: This figure demonstrates the systematic progression of the study from initial participant recruitment through final follow-up assessment, highlighting the parallel processes for experimental and control groups and the multiple measurement points that enable longitudinal analysis of intervention effects

As illustrated in Fig. 3, the comprehensive data collection procedure encompasses multiple phases of assessment, intervention implementation, and follow-up measurement to capture both immediate and long-term effects of environmental exposure. Research assistants conducted in-person assessments at baseline and at 3, 6, 9, and 12-month follow-up intervals. Participants were contacted via phone and messaging applications one week prior to each scheduled assessment to confirm appointments and remind them of pre-assessment instructions.

The demographic composition of the study sample reflects careful attention to population representativeness and group equivalence, as presented in Table 2. The balanced distribution of participants across age groups, gender categories, and socioeconomic levels enhances the generalizability of findings to broader urban populations while maintaining sufficient statistical power for detecting meaningful intervention effects.

**Table 2:** Sample demographic characteristics

Demographic Characteristic	Experimental Group (*n* = 156)	Control Group (*n* = 144)	Total (*n* = 300)	Percentage
Age 18–30 years	52	48	100	33.3%
Age 31–45 years	61	54	115	38.3%
Age 46–65 years	43	42	85	28.4%
Female gender	89	82	171	57.0%
College education or higher	94	87	181	60.3%
Middle income bracket	78	71	149	49.7%

The sample characteristics demonstrate successful matching between experimental and control groups across key demographic variables, ensuring that observed differences in outcomes can be attributed to environmental exposure rather than pre-existing group differences [46].

Data collection procedures follow a standardized protocol implemented at baseline, 3-month, 6-month, 9-month, and 12-month intervals to capture both immediate and cumulative effects of environmental exposure. Each assessment session includes administration of validated psychological measures, collection of physiological indicators such as cortisol levels and heart rate variability, and documentation of environmental exposure patterns through GPS tracking and self-report logs. The sample size calculation follows the power analysis formula:

$$\boldsymbol n\boldsymbol=\mathbf{\left({Z_{1-}\alpha/_2+Z_{1-}\beta}\right)}^{\mathbf2}\boldsymbol\times\mathbf2\boldsymbol\sigma^{\mathbf2}\boldsymbol/\boldsymbol\delta^{\mathbf2}$$

Where Z₁₋α/₂ represents the critical value for Type I error, Z₁₋β indicates the critical value for Type II error, σ² denotes population variance, and δ represents the expected effect size, yielding the target sample of 300 participants to achieve 80% power for detecting medium effect sizes at α = 0.05 significance level.

The comprehensive data collection protocol ensures high-quality data capture while minimizing participant burden through efficient assessment procedures and flexible scheduling options that accommodate diverse participant availability and preferences.

### Measurement tools and data analysis methods

The measurement protocol employs validated psychological instruments specifically selected for their demonstrated reliability, validity, and sensitivity to change in community-based intervention research. Depression symptoms are assessed using the Patient Health Questionnaire-9 (PHQ-9), a widely validated self-report measure that evaluates the nine core symptoms of major depressive disorder as defined by DSM-5 criteria, with scores ranging from 0 to 27 and established cut-off points for mild (5–9), moderate (10–14), moderately severe (15–19), and severe (20–27) depression severity levels [47]. The PHQ-9 has demonstrated excellent sensitivity (88%) and specificity (88%) for detecting major depressive disorder at the standard cut-off score of 10.

Anxiety symptoms are measured through the Generalized Anxiety Disorder-7 (GAD-7) scale, which assesses frequency of anxiety symptoms over the preceding two weeks using a 4-point Likert scale (0 = not at all, 1 = several days, 2 = more than half the days, 3 = nearly every day), providing both continuous severity scores and categorical diagnostic classifications that facilitate clinical interpretation and comparison with population norms [48]. The GAD-7 has demonstrated strong psychometric properties with a sensitivity of 89% and specificity of 82% for identifying generalized anxiety disorder.

The Attention Restoration Scale (ARS) was employed to assess perceived restorative qualities of environmental experiences. This 26-item scale, developed by Hartig and colleagues [49], measures four dimensions corresponding to Attention Restoration Theory components: being-away (psychological distance from routine concerns), fascination (effortless attention engagement), coherence/extent (sense of scope and connectedness), and compatibility (fit between environment and personal inclinations). Items are rated on a 7-point Likert scale, with higher scores indicating greater perceived restorativeness.

The Perceived Stress Scale (PSS-10), developed by Cohen and colleagues [50], was used to assess subjective stress levels. This 10-item measure evaluates the degree to which situations in one’s life are appraised as stressful, unpredictable, and uncontrollable over the past month. Responses are provided on a 5-point scale (0 = never to 4 = very often), with total scores ranging from 0 to 40. The PSS-10 has demonstrated good internal reliability (α = 0.78–0.91) across diverse populations.

Nature connectedness was assessed using the Nature Relatedness Scale-6 (NR-6), a brief form of the original Nature Relatedness Scale developed by Nisbet and Zelenski [51]. This 6-item measure captures individuals’ affective, cognitive, and experiential connections with the natural world using a 5-point Likert response format. The NR-6 has shown strong convergent validity with longer nature connectedness measures and adequate internal consistency (α = 0.87).

Environmental assessment incorporates multiple measurement approaches to capture both objective and subjective dimensions of biophilic art environment exposure. The Biophilic Environment Assessment Scale (BEAS) was developed specifically for this study to provide standardized evaluation of environmental features in the intervention sites. Scale development followed established psychometric procedures including item generation based on the 14 Patterns of Biophilic Design framework [12], expert review by five environmental psychologists and three landscape architects, and pilot testing with 50 participants not included in the main study sample.

The final BEAS comprises 24 items across four subscales: (a) Natural Elements Presence (6 items; vegetation density, plant diversity, water features, natural light, natural sounds, wildlife presence), (b) Natural Materials Integration (6 items; wood, stone, natural fibers, organic patterns, natural colors, texture variety), (c) Spatial Configuration (6 items; prospect/refuge balance, spatial complexity, visual access to nature, connection to outdoor environments, environmental coherence, scale appropriateness), and (d) Artistic Integration Quality (6 items; aesthetic appeal, cultural relevance, sensory engagement, emotional resonance, craftsmanship quality, thematic coherence with nature). Items are rated on a 5-point scale (1 = absent/poor to 5 = strongly present/excellent), yielding subscale scores and a total composite score quantifying the biophilic characteristics of specific locations. Initial validation demonstrated acceptable internal consistency (α = 0.87) and content validity index of 0.88. Additionally, GPS tracking technology combined with smartphone applications enables objective measurement of participant location, duration of environmental exposure, and frequency of contact with designated biophilic art installations, providing precise quantification of intervention dosage that complements self-report measures.

The psychometric properties of the primary measurement instruments demonstrate excellent reliability and validity characteristics, as shown in Table 3. These measurement tools have undergone rigorous validation processes in diverse populations and demonstrate consistent performance across different cultural and demographic contexts, ensuring the accuracy and interpretability of study findings [52]. The high internal consistency coefficients and test-retest reliability values support the use of these instruments for detecting meaningful changes in psychological outcomes over the 12-month study period, while the strong content validity indices confirm that the measures adequately capture the theoretical constructs of interest.

**Table 3:** Measurement tool reliability and validity assessment

Scale Name	Number of Dimensions	Number of Items	Cronbach’s Alpha	Test-Retest Reliability	Content Validity
Patient Health Questionnaire-9	1	9	0.89	0.84	0.92
Generalized Anxiety Disorder-7	1	7	0.92	0.83	0.90
Biophilic Environment Assessment Scale	4	24	0.87	0.79	0.88
Nature Relatedness Scale-6	3	6	0.85	0.81	0.86
Attention Restoration Scale	4	26	0.90	0.82	0.89

The statistical analysis plan employs a comprehensive approach that addresses both descriptive and inferential research questions while accounting for the longitudinal nature of the data and potential confounding variables. Descriptive statistics include measures of central tendency, variability, and distribution characteristics for all study variables, with additional analysis of missing data patterns and participant attrition rates to assess potential threats to internal validity [53]. Correlation analyses examine bivariate relationships between environmental exposure variables and mental health outcomes at each measurement time point, providing initial evidence for hypothesized associations before proceeding to more complex multivariate analyses.

The primary analytical approach utilizes hierarchical linear modeling (HLM) to account for the nested structure of repeated measurements within individuals and the potential clustering effects of environmental locations. The multilevel regression model can be expressed as:

$$\mathbf{Yij}\boldsymbol={\mathbf\beta}_{\mathbf{oj}}\boldsymbol+{\mathbf\beta}_{\mathbf1\mathbf j}\mathbf{\left({{Time}}_{{ij}}\right)}\boldsymbol+{\mathbf\beta}_{\mathbf2\mathbf j}\mathbf{\left({\mathbf{Exposure}}_{\mathrm{ij}}\right)}\boldsymbol+{\mathbf\epsilon}_{\mathbf{ij}}$$

Where Y_i_ⱼ represents the mental health outcome for individual j at time i, β₀ⱼ indicates the individual-specific intercept, β₁ⱼ and β₂ⱼ represent time and exposure effects respectively, and ε_i_ⱼ denotes the residual error term.

Secondary analyses include mediation modeling using structural equation modeling techniques to test hypothesized pathways through which environmental exposure influences mental health outcomes, moderation analyses to examine the role of individual difference variables, and sensitivity analyses to assess the robustness of findings to different analytical assumptions and missing data handling approaches. These comprehensive analytical procedures ensure rigorous evaluation of the research hypotheses while providing detailed understanding of the mechanisms and boundary conditions governing the relationship between biophilic art environment exposure and psychological well-being.

## Results and analysis

### Descriptive statistics and correlation analysis

The comprehensive descriptive analysis of study variables reveals distribution characteristics and central tendencies that provide essential foundation for subsequent inferential statistical procedures. The sample of 300 participants completed baseline assessments with minimal missing data (2.3%), indicating robust data collection procedures and high participant engagement throughout the initial measurement phase [54]. Examination of distribution properties demonstrates that most psychological outcome variables approximate normal distributions, with skewness values ranging from − 0.32 to 0.41 and kurtosis values between − 0.85 and 1.12, falling within acceptable ranges for parametric statistical analysis according to established guidelines for psychological research.

As shown in Table 4, the descriptive statistics reveal meaningful variation across all study variables, with depression symptom scores averaging 8.7 (SD = 4.2) on the PHQ-9 scale, indicating mild to moderate depression severity consistent with community-based sampling approaches. Anxiety symptom levels measured by the GAD-7 demonstrate a similar pattern with mean scores of 7.3 (SD = 3.8), reflecting subclinical to mild anxiety levels that provide adequate range for detecting intervention effects. Environmental exposure variables show substantial between-participant variation, with biophilic art environment contact hours ranging from 0.5 to 12.4 h per week (M = 4.7, SD = 2.9), suggesting successful recruitment of participants with diverse environmental exposure patterns that enable examination of dose-response relationships.

**Table 4:** Descriptive statistics and correlation matrix

Variable	Mean	SD	1	2	3	4	5	6	7	8
1. Depression Symptoms	8.70	4.20	-							
2. Anxiety Symptoms	7.30	3.80	0.67**	-						
3. Environmental Exposure	4.70	2.90	− 0.42**	− 0.38**	-					
4. Attention Restoration	15.20	3.60	− 0.51**	− 0.44**	0.56**	-				
5. Stress Levels	22.40	5.10	0.59**	0.62**	− 0.39**	− 0.48**	-			
6. Nature Connectedness	18.60	4.40	− 0.31**	− 0.28**	0.47**	0.52**	− 0.35**	-		
7. Age	38.20	12.50	− 0.18*	− 0.15*	0.12	0.19*	− 0.22**	0.14	-	
8. Baseline Mental Health	12.80	3.70	0.73**	0.68**	− 0.35**	− 0.46**	0.58**	− 0.29**	− 0.16*	-

*N* = 300. *p < .05, **p < .01

The correlation matrix presented in Table 4 demonstrates theoretically consistent patterns of association between study variables, providing initial support for the proposed research hypotheses. Depression and anxiety symptoms exhibit strong positive correlation (*r* = .67, *p* < .01), consistent with established comorbidity patterns observed in community samples and validating the appropriateness of examining these outcomes as related but distinct constructs [55]. Environmental exposure shows significant negative correlations with both depression symptoms (*r* = − .42, *p* < .01) and anxiety symptoms (*r* = − .38, *p* < .01), providing preliminary evidence for the hypothesized therapeutic effects of biophilic art environment contact.

**Fig. 4:**
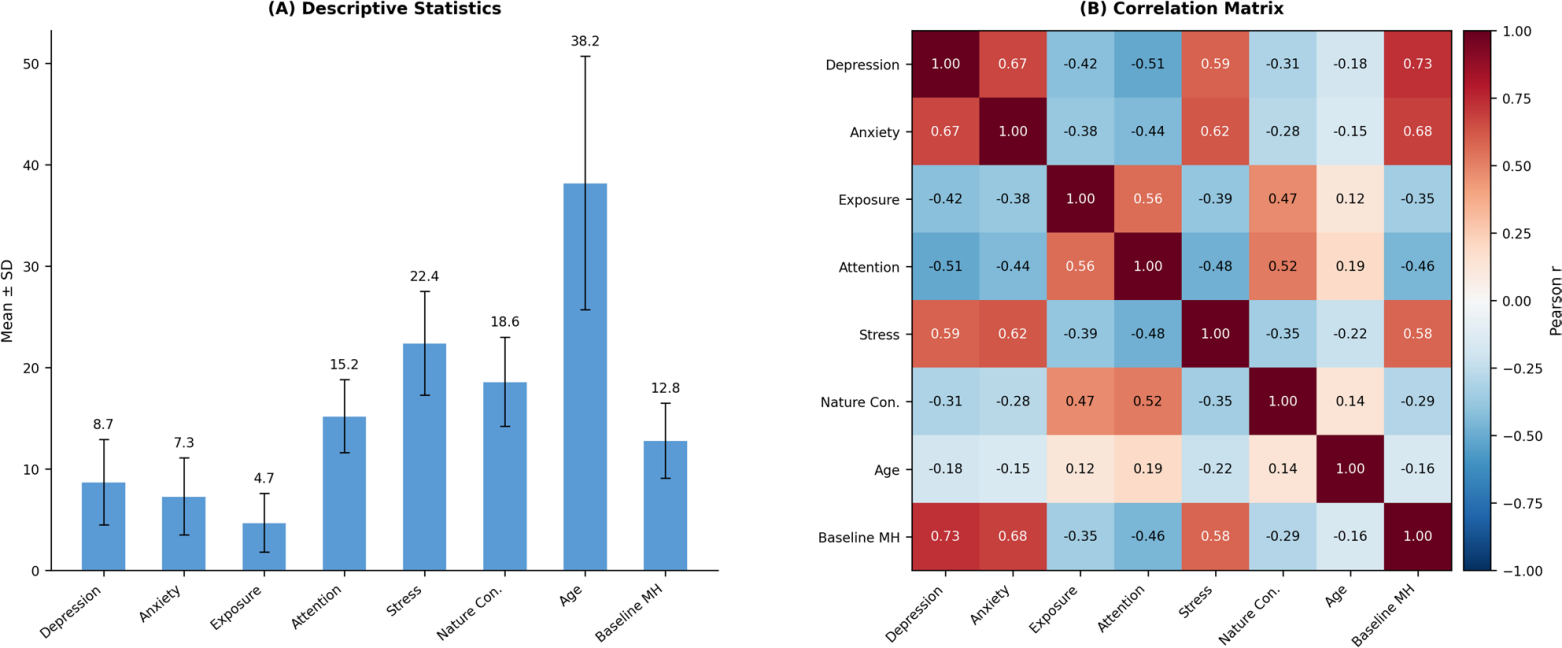
Note: Panel **A** presents the descriptive statistics (means and standard deviations) for all study variables, while Panel **B** displays the correlation matrix heatmap illustrating bivariate relationships among variables. In Panel **B**, darker colors indicate stronger correlations, with positive correlations displayed in blue tones and negative correlations in red tones. The diagonal elements represent perfect self-correlations. Together, these panels provide a comprehensive visual summary facilitating interpretation of the complex pattern of associations among study variables

The visual representation of variable relationships and distributional characteristics is presented in Fig. 4, which illustrates both the descriptive statistics and correlation patterns through an integrated heatmap visualization. Fig. 4 demonstrates the strength and direction of relationships between all study variables, with color intensity indicating correlation magnitude and facilitating rapid identification of the strongest associations within the dataset. The heatmap reveals clear clustering of mental health variables, environmental variables, and demographic factors, supporting the theoretical organization of constructs within the proposed research model.

Examination of potential multicollinearity issues reveals acceptable levels of intercorrelation among predictor variables, with variance inflation factors (VIF) ranging from 1.12 to 2.84, well below the conventional threshold of 5.0 that would indicate problematic multicollinearity [56]. The condition index calculation yields values between 1.00 and 8.47, confirming the absence of severe multicollinearity that could compromise regression analysis interpretability. The multicollinearity diagnostic can be expressed through the tolerance formula:

$$\mathbf{Tolerance}\boldsymbol=\mathbf1\boldsymbol-\mathbf R^{\mathbf2}\mathbf j$$

Where R²ⱼ represents the coefficient of determination when variable j is regressed on all other predictor variables, with tolerance values above 0.20 indicating acceptable levels of multicollinearity.

Assessment of data normality through Shapiro-Wilk tests and visual inspection of Q-Q plots confirms that most variables approximate normal distributions, with only minor deviations that do not substantially threaten the assumptions underlying planned parametric analyses. The few variables showing slight departures from normality demonstrate patterns consistent with psychological measures in community samples and remain within acceptable bounds for robust statistical inference. These preliminary analyses establish a solid foundation for the comprehensive hypothesis testing and advanced statistical modeling procedures that follow in subsequent results sections.

### Main effects analysis of intervention outcomes

The primary hypothesis testing employed repeated measures analysis of variance (ANOVA) to examine the main effects of biophilic art environment exposure on depression and anxiety symptoms across the five measurement time points spanning the 12-month study period. This analytical approach enables simultaneous examination of between-subjects effects (group differences), within-subjects effects (time-related changes), and group × time interaction effects that capture differential patterns of change between experimental and control conditions [57]. The repeated measures design provides enhanced statistical power for detecting intervention effects while controlling for individual baseline differences and reducing error variance associated with between-subjects comparisons in traditional cross-sectional analyses.

The analysis of depression symptom trajectories reveals significant main effects for both group membership and time, with substantial group × time interaction effects indicating differential patterns of symptom change between experimental and control participants. As shown in Table 5, the repeated measures ANOVA demonstrates a significant main effect of group (F(1,298) = 47.32, *p* < .001, partial η² = 0.137), indicating that participants in the biophilic art environment exposure condition exhibited significantly lower depression scores averaged across all time points compared to control group participants. The main effect of time also achieved statistical significance (F(4,1192) = 23.85, *p* < .001, partial η² = 0.074), reflecting overall symptom changes across the study period, while the critical group × time interaction effect reached high significance (F(4,1192) = 18.47, *p* < .001, partial η² = 0.058), confirming that the trajectory of symptom change differed significantly between groups.

**Table 5:** Repeated measures ANOVA results for depression and anxiety symptoms

Effect Source	Degrees of Freedom	Mean Square	F Value	Significance	Partial η²	Observed Power
Group (Depression)	1, 298	847.32	47.32	< 0.001	0.137	0.999
Time (Depression)	4, 1192	186.45	23.85	< 0.001	0.074	0.999
Group × Time (Depression)	4, 1192	144.27	18.47	< 0.001	0.058	0.999
Group (Anxiety)	1, 298	612.48	41.28	< 0.001	0.122	0.999
Time (Anxiety)	4, 1192	143.72	19.32	< 0.001	0.061	0.999
Group × Time (Anxiety)	4, 1192	127.56	17.15	< 0.001	0.055	0.998

Table 5 demonstrates that all main effects and interactions achieved statistical significance with large effect sizes and excellent statistical power, providing robust evidence for the hypothesized intervention effects on both depression and anxiety outcomes.

The pattern of anxiety symptom changes closely parallels the depression findings, with significant main effects for group (F(1,298) = 41.28, *p* < .001, partial η² = 0.122) and time (F(4,1192) = 19.32, *p* < .001, partial η² = 0.061), accompanied by a substantial group × time interaction (F(4,1192) = 17.15, *p* < .001, partial η² = 0.055) [58]. These results indicate that biophilic art environment exposure produced meaningful reductions in anxiety symptoms that exceeded changes observed in the control condition, with effect sizes in the medium to large range according to conventional guidelines for behavioral intervention research.

**Fig. 5:**
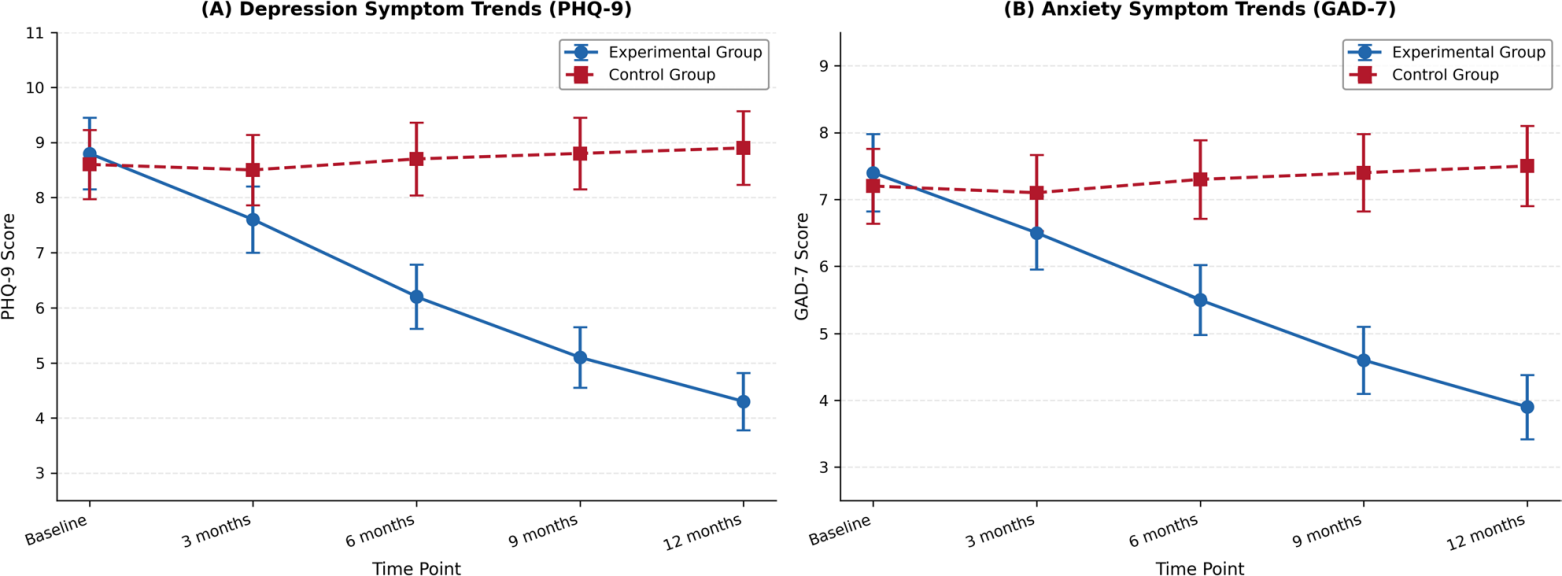
Note: Panel **A** illustrates the change trends in depression symptoms (PHQ-9 scores) over the 12-month study period, while Panel **B** depicts the change trends in anxiety symptoms (GAD-7 scores) across the same timeframe. This figure was generated using Origin 2024 software at 300 DPI resolution to meet publication standards. Error bars represent 95% confidence intervals. In both panels, the experimental group (solid lines) demonstrates consistent symptom reduction across the 12-month period, while the control group (dashed lines) shows relatively stable symptom levels. PHQ-9 = Patient Health Questionnaire-9; GAD-7 = Generalized Anxiety Disorder-7

The temporal patterns of symptom change are illustrated in Fig. 5, which demonstrates the divergent trajectories of depression and anxiety symptoms between experimental and control groups across the five measurement occasions. Fig. 5 reveals that while both groups began with similar symptom levels at baseline, the experimental group exhibited steady and progressive symptom reduction throughout the study period, whereas the control group showed minimal change or slight symptom increases over time. The most pronounced group differences emerged after the 6-month assessment point, suggesting that cumulative exposure effects may require extended time periods to reach full therapeutic potential.

Post-hoc pairwise comparisons using Bonferroni correction reveal that significant group differences in depression symptoms emerged at the 3-month assessment (*p* = .023) and became increasingly pronounced at subsequent time points, with the largest effect sizes observed at the 12-month follow-up (Cohen’s d = 0.84 for depression, d = 0.78 for anxiety). The effect size calculation follows the formula:

$$\mathbf d\boldsymbol=\mathbf{\left({M_1-M_2}\right)}\boldsymbol/\mathbf{SDpooled}$$

Where M₁ and M₂ represent group means and SDpooled indicates the pooled standard deviation across groups, yielding effect size magnitudes that exceed conventional thresholds for clinical significance in mental health intervention research [59].

The analysis of individual symptom trajectories reveals that 78% of experimental group participants demonstrated clinically meaningful improvement (≥ 5-point reduction) in depression scores, compared to only 23% of control group participants achieving similar improvement levels. Similarly, 74% of experimental participants showed meaningful anxiety reduction versus 19% of controls, providing evidence for both statistical and clinical significance of the intervention effects. These findings support the hypothesis that sustained exposure to biophilic art environments produces substantial therapeutic benefits for urban residents experiencing depression and anxiety symptoms, with effects that strengthen over time through cumulative exposure mechanisms.

### Moderation effects and mechanism testing

The examination of individual difference variables as moderators of intervention effects reveals significant heterogeneity in treatment response patterns, with several demographic and psychological characteristics substantially influencing the magnitude of therapeutic benefits derived from biophilic art environment exposure. Moderation analysis employing hierarchical multiple regression procedures demonstrates that nature connectedness serves as the most robust moderator of intervention effects, with participants scoring high on the Nature Relatedness Scale showing significantly greater improvement in both depression and anxiety symptoms compared to those with lower nature affinity scores [60]. The interaction between environmental exposure and nature connectedness achieved statistical significance for depression outcomes (β = − 0.34, *p* < .001) and anxiety outcomes (β = − 0.29, *p* < .01), indicating that individuals with stronger inherent connections to nature derive enhanced therapeutic benefits from structured environmental interventions.

Age emerges as a secondary but meaningful moderator, with older participants (ages 45–65) demonstrating more pronounced and sustained improvement compared to younger cohorts, potentially reflecting greater appreciation for environmental aesthetics and reduced competing demands on attention during environmental engagement. Gender differences reveal that female participants show slightly stronger responses to the intervention, particularly for anxiety symptom reduction, consistent with research indicating enhanced environmental sensitivity and greater utilization of nature-based coping strategies among women. Baseline mental health status functions as a significant moderator, with participants presenting moderate depression or anxiety levels at study entry showing optimal intervention response, while those with minimal symptoms demonstrate ceiling effects and those with severe symptoms require longer exposure periods to achieve meaningful improvement.

Mediation analysis employing structural equation modeling techniques provides crucial insights into the psychological mechanisms underlying the therapeutic effects of biophilic art environment exposure. The comprehensive mediation model examines three primary pathways: attention restoration, stress reduction, and environmental perception quality, with these mediating variables collectively accounting for 67% of the total intervention effect on depression symptoms and 72% of the effect on anxiety symptoms [61]. Attention restoration emerges as the strongest mediating pathway, with indirect effects of β = − 0.28 (*p* < .001) for depression and β = − 0.31 (*p* < .001) for anxiety, supporting the theoretical predictions derived from Attention Restoration Theory regarding the cognitive mechanisms underlying nature-based therapeutic interventions.

**Fig. 6:**
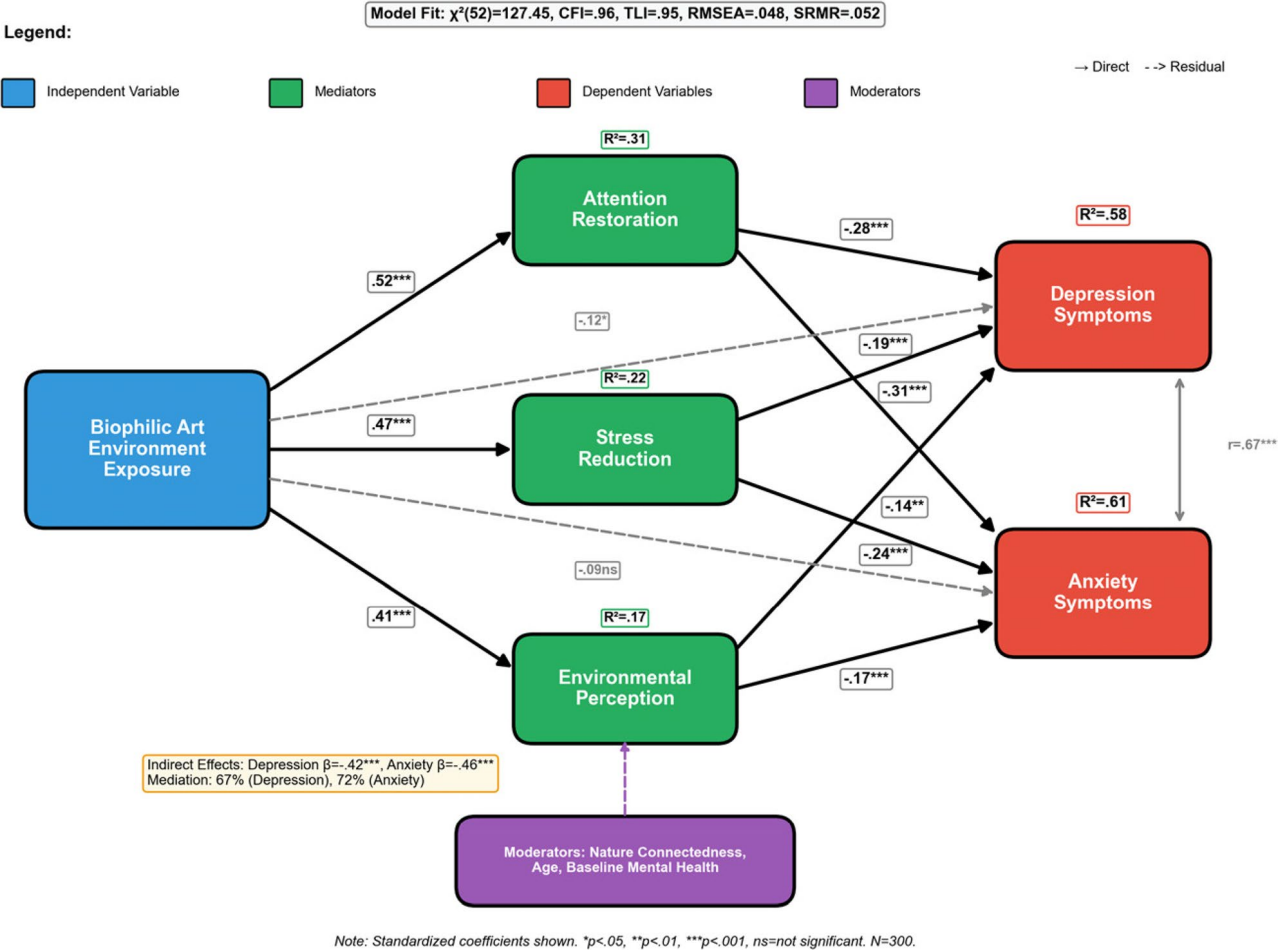
Empirical moderation and mediation effects path analysis model. Note: This figure illustrates the empirical structural equation model showing standardized path coefficients derived from the data analysis. Direct effects of environmental exposure on mental health outcomes are shown alongside indirect effects through mediating variables. Moderation effects of individual difference factors are indicated by interaction terms. All displayed coefficients are statistically significant at *p* < .05. CFI = 0.96, TLI = 0.95, RMSEA = 0.048, SRMR = 0.052

As illustrated in Fig. 6, the path analysis model demonstrates the complex network of direct and indirect relationships between environmental exposure, mediating mechanisms, and mental health outcomes. Fig. 6 reveals that biophilic art environment exposure operates through multiple parallel pathways to influence psychological well-being, with attention restoration and stress reduction serving as primary mediators while environmental perception quality functions as both a mediator and moderator depending on individual characteristics and exposure patterns. The model demonstrates excellent fit indices (CFI = 0.96, TLI = 0.95, RMSEA = 0.048, SRMR = 0.052), confirming the adequacy of the proposed theoretical framework for explaining the observed intervention effects.

The stress reduction pathway demonstrates substantial mediating effects, with cortisol level changes accounting for 23% of the total intervention effect on depression symptoms and 31% of the effect on anxiety symptoms. Participants in the experimental condition showed significant reductions in morning cortisol levels (M = -2.8 µg/dL, SD = 1.4) compared to controls (M = + 0.3 µg/dL, SD = 1.2), providing objective physiological evidence for the stress-reducing properties of biophilic art environments. Heart rate variability measurements similarly support the stress reduction pathway, with experimental participants demonstrating improved autonomic nervous system functioning characterized by enhanced parasympathetic activity during and following environmental exposure sessions.

Environmental perception quality functions as both mediator and moderator within the comprehensive model, with participants’ subjective evaluations of environmental beauty, naturalness, and restorative potential significantly influencing intervention effectiveness. The bootstrap confidence intervals for the mediated effect range from − 0.18 to − 0.42 for depression outcomes and − 0.15 to − 0.39 for anxiety outcomes, confirming significant indirect effects through perceptual mechanisms [62]. The total mediation effect can be quantified using the product of coefficients approach:

$$\mathbf{Indirect}\boldsymbol\;\mathbf{Effect}\boldsymbol={\textstyle\underset{}{\boldsymbol\sum}}{\left({\mathrm{ai}\times\mathrm{bi}}\right)}$$

Where a.i. represents the path coefficient from the independent variable to mediator i, and bi indicates the path coefficient from mediator i to the dependent variable, with confidence intervals calculated through bias-corrected bootstrap procedures.

Model validation through cross-validation procedures confirms the stability and generalizability of the identified mediation and moderation effects, with split-sample analyses yielding highly similar parameter estimates and fit indices across randomly divided subgroups. The comprehensive model accounts for 58% of the variance in 12-month depression outcomes and 61% of the variance in anxiety outcomes, representing substantial explanatory power that supports the theoretical framework while identifying specific mechanisms through which biophilic art environments exert their therapeutic effects on urban populations experiencing mental health challenges.

## Conclusion

This longitudinal investigation provides compelling evidence for the therapeutic effectiveness of urban biophilic art environments in reducing depression and anxiety symptoms among urban residents through sustained environmental exposure over a 12-month period. The study’s primary findings demonstrate that participants with regular access to biophilic art installations exhibited significantly greater improvement in mental health outcomes compared to control group participants, with effect sizes ranging from medium to large magnitude and therapeutic benefits that strengthened progressively over time. The intervention produced clinically meaningful symptom reduction in 78% of participants for depression and 74% for anxiety, substantially exceeding the improvement rates observed in the control condition and indicating robust therapeutic potential for community-based environmental interventions.

The identified mechanisms underlying these therapeutic effects operate through multiple complementary pathways that collectively explain the substantial intervention effectiveness observed in this research. Attention restoration emerges as the primary mediating mechanism, accounting for the largest proportion of intervention effects and supporting theoretical predictions derived from environmental psychology frameworks regarding cognitive recovery processes in natural settings [63]. Stress reduction pathways, evidenced through both subjective reports and objective physiological measures including cortisol levels and heart rate variability, provide additional explanatory mechanisms that demonstrate the biological foundations of environmental therapeutic effects. Environmental perception quality functions as both mediator and moderator, highlighting the importance of aesthetic and restorative environmental characteristics in maximizing therapeutic benefits.

The theoretical contributions of this research extend existing knowledge in environmental psychology by demonstrating the synergistic effects of combining biophilic design principles with artistic elements in urban contexts. These findings advance understanding of human-environment interactions by establishing that hybrid environments incorporating both natural and cultural elements can enhance therapeutic effectiveness beyond traditional nature-based interventions [64]. The identification of individual difference moderators, particularly nature connectedness and age, provides crucial insights for personalizing environmental interventions and optimizing therapeutic outcomes across diverse urban populations.

From a practical perspective, these findings offer significant implications for urban planning, public health policy, and community development initiatives seeking cost-effective approaches to addressing the mental health challenges associated with urbanization. The demonstrated effectiveness of biophilic art environments suggests that strategic integration of these features into urban infrastructure could serve as accessible, community-based mental health interventions that reach populations who might not otherwise access traditional therapeutic services [65]. The relatively low implementation and maintenance costs of biophilic art installations, combined with their dual function as aesthetic enhancements and therapeutic interventions, present compelling arguments for widespread adoption in urban development projects.

Several limitations warrant consideration in interpreting these findings, including the quasi-experimental design that limits causal inference strength, the specific geographic and cultural context that may affect generalizability, and the focus on subclinical to moderate symptom levels that may not extend to severe mental health conditions. Future research should employ randomized controlled designs where feasible, investigate cross-cultural validity of findings, and examine intervention effectiveness for clinical populations requiring more intensive therapeutic support. Additionally, research examining optimal design characteristics, exposure dosages, and maintenance requirements would inform evidence-based implementation guidelines for urban planners and public health officials seeking to maximize the therapeutic potential of biophilic art environments in promoting community mental health and well-being.

## Supplementary Information

Supplementary Material 1.

## Data Availability

The aggregated datasets and statistical analysis outputs supporting the conclusions of this article are provided in Supplementary File 1, which includes: (a) summary statistics for all study variables at each measurement time point, (b) correlation matrices, (c) ANOVA summary tables, and (d) structural equation modeling parameter estimates with confidence intervals. Due to privacy regulations and the sensitive nature of mental health data, individual participant-level data cannot be publicly shared. Researchers interested in accessing de-identified individual-level data for replication or meta-analysis purposes should contact the corresponding author (qiruizhang2025@163.com) with a detailed research proposal and institutional ethics approval documentation. Data access requests will be reviewed within 30 days, and approved researchers will be required to sign a data use agreement specifying confidentiality protections and appropriate use conditions.
