# Supplementary material for: Long-term effects of urban biophilic art environments on depression and anxiety symptoms: a longitudinal intervention study
Source: BMC Psychol. 2026 Mar 4;14:500. doi: 10.1186/s40359-026-04262-6 (PMC13067697; doi:10.1186/s40359-026-04262-6)
Supplement: Supplementary file 1 — Supplementary Material 1. [file 40359_2026_4262_MOESM1_ESM.docx]

**Supplementary File 1**

**Long-term Effects of Urban Biophilic Art Environments on Depression and Anxiety Symptoms: A Longitudinal Intervention Study**

**Table S1. Summary Statistics for Depression Symptoms (PHQ-9) by Group and Time Point**

| **Time Point** | **Group** | **n** | **Mean** | **SD** | **95% CI Lower** | **95% CI Upper** | **Min** | **Max** | **Skewness** | **Kurtosis** |
| --- | --- | --- | --- | --- | --- | --- | --- | --- | --- | --- |
| Baseline | Experimental | 156 | 8.82 | 4.15 | 8.16 | 9.48 | 1 | 19 | 0.34 | -0.42 |
| Baseline | Control | 144 | 8.57 | 4.26 | 7.87 | 9.27 | 0 | 20 | 0.38 | -0.38 |
| 3-Month | Experimental | 152 | 7.45 | 3.89 | 6.83 | 8.07 | 0 | 17 | 0.29 | -0.51 |
| 3-Month | Control | 141 | 8.62 | 4.31 | 7.90 | 9.34 | 1 | 19 | 0.36 | -0.45 |
| 6-Month | Experimental | 149 | 6.12 | 3.52 | 5.55 | 6.69 | 0 | 15 | 0.22 | -0.63 |
| 6-Month | Control | 138 | 8.78 | 4.38 | 8.04 | 9.52 | 1 | 20 | 0.41 | -0.32 |
| 9-Month | Experimental | 147 | 5.28 | 3.21 | 4.76 | 5.80 | 0 | 14 | 0.18 | -0.71 |
| 9-Month | Control | 136 | 8.91 | 4.45 | 8.15 | 9.67 | 1 | 21 | 0.44 | -0.28 |
| 12-Month | Experimental | 145 | 4.67 | 2.98 | 4.18 | 5.16 | 0 | 13 | 0.15 | -0.78 |
| 12-Month | Control | 134 | 9.12 | 4.52 | 8.35 | 9.89 | 1 | 21 | 0.47 | -0.24 |

**Table S2. Summary Statistics for Anxiety Symptoms (GAD-7) by Group and Time Point**

| **Time Point** | **Group** | **n** | **Mean** | **SD** | **95% CI Lower** | **95% CI Upper** | **Min** | **Max** | **Skewness** | **Kurtosis** |
| --- | --- | --- | --- | --- | --- | --- | --- | --- | --- | --- |
| Baseline | Experimental | 156 | 7.38 | 3.74 | 6.79 | 7.97 | 0 | 17 | 0.31 | -0.48 |
| Baseline | Control | 144 | 7.21 | 3.87 | 6.57 | 7.85 | 0 | 18 | 0.35 | -0.44 |
| 3-Month | Experimental | 152 | 6.34 | 3.48 | 5.78 | 6.90 | 0 | 15 | 0.26 | -0.55 |
| 3-Month | Control | 141 | 7.35 | 3.92 | 6.70 | 8.00 | 0 | 17 | 0.33 | -0.49 |
| 6-Month | Experimental | 149 | 5.21 | 3.15 | 4.70 | 5.72 | 0 | 14 | 0.19 | -0.67 |
| 6-Month | Control | 138 | 7.52 | 4.01 | 6.84 | 8.20 | 0 | 18 | 0.38 | -0.41 |
| 9-Month | Experimental | 147 | 4.45 | 2.89 | 3.98 | 4.92 | 0 | 12 | 0.14 | -0.74 |
| 9-Month | Control | 136 | 7.68 | 4.08 | 6.99 | 8.37 | 1 | 19 | 0.42 | -0.36 |
| 12-Month | Experimental | 145 | 3.92 | 2.67 | 3.48 | 4.36 | 0 | 11 | 0.11 | -0.81 |
| 12-Month | Control | 134 | 7.85 | 4.15 | 7.14 | 8.56 | 1 | 19 | 0.45 | -0.31 |

**Table S3. Summary Statistics for Attention Restoration Scale by Group and Time Point**

| **Time Point** | **Group** | **n** | **Mean** | **SD** | **95% CI Lower** | **95% CI Upper** |
| --- | --- | --- | --- | --- | --- | --- |
| Baseline | Experimental | 156 | 15.12 | 3.58 | 14.55 | 15.69 |
| Baseline | Control | 144 | 15.29 | 3.63 | 14.69 | 15.89 |
| 3-Month | Experimental | 152 | 17.45 | 3.42 | 16.90 | 18.00 |
| 3-Month | Control | 141 | 15.38 | 3.71 | 14.76 | 16.00 |
| 6-Month | Experimental | 149 | 19.28 | 3.25 | 18.75 | 19.81 |
| 6-Month | Control | 138 | 15.42 | 3.78 | 14.78 | 16.06 |
| 9-Month | Experimental | 147 | 20.67 | 3.12 | 20.16 | 21.18 |
| 9-Month | Control | 136 | 15.51 | 3.82 | 14.86 | 16.16 |
| 12-Month | Experimental | 145 | 21.84 | 2.98 | 21.35 | 22.33 |
| 12-Month | Control | 134 | 15.58 | 3.89 | 14.91 | 16.25 |

**Table S4. Summary Statistics for Perceived Stress Scale by Group and Time Point**

| **Time Point** | **Group** | **n** | **Mean** | **SD** | **95% CI Lower** | **95% CI Upper** |
| --- | --- | --- | --- | --- | --- | --- |
| Baseline | Experimental | 156 | 22.35 | 5.08 | 21.55 | 23.15 |
| Baseline | Control | 144 | 22.46 | 5.13 | 21.61 | 23.31 |
| 3-Month | Experimental | 152 | 20.18 | 4.72 | 19.42 | 20.94 |
| 3-Month | Control | 141 | 22.58 | 5.21 | 21.71 | 23.45 |
| 6-Month | Experimental | 149 | 18.24 | 4.35 | 17.53 | 18.95 |
| 6-Month | Control | 138 | 22.71 | 5.28 | 21.82 | 23.60 |
| 9-Month | Experimental | 147 | 16.85 | 4.12 | 16.18 | 17.52 |
| 9-Month | Control | 136 | 22.89 | 5.34 | 21.98 | 23.80 |
| 12-Month | Experimental | 145 | 15.72 | 3.95 | 15.07 | 16.37 |
| 12-Month | Control | 134 | 23.05 | 5.41 | 22.12 | 23.98 |

**Table S5. Summary Statistics for Salivary Cortisol Levels (μg/dL) by Group and Time Point**

| **Time Point** | **Group** | **n** | **Mean** | **SD** | **95% CI Lower** | **95% CI Upper** |
| --- | --- | --- | --- | --- | --- | --- |
| Baseline | Experimental | 156 | 14.82 | 3.45 | 14.27 | 15.37 |
| Baseline | Control | 144 | 14.76 | 3.52 | 14.18 | 15.34 |
| 3-Month | Experimental | 152 | 13.65 | 3.28 | 13.12 | 14.18 |
| 3-Month | Control | 141 | 14.89 | 3.58 | 14.29 | 15.49 |
| 6-Month | Experimental | 149 | 12.78 | 3.12 | 12.27 | 13.29 |
| 6-Month | Control | 138 | 15.02 | 3.64 | 14.41 | 15.63 |
| 9-Month | Experimental | 147 | 12.15 | 2.98 | 11.66 | 12.64 |
| 9-Month | Control | 136 | 15.18 | 3.71 | 14.55 | 15.81 |
| 12-Month | Experimental | 145 | 12.02 | 2.85 | 11.55 | 12.49 |
| 12-Month | Control | 134 | 15.06 | 3.68 | 14.43 | 15.69 |

**Table S6. Summary Statistics for Heart Rate Variability (RMSSD, ms) by Group and Time Point**

| **Time Point** | **Group** | **n** | **Mean** | **SD** | **95% CI Lower** | **95% CI Upper** |
| --- | --- | --- | --- | --- | --- | --- |
| Baseline | Experimental | 156 | 32.45 | 8.72 | 31.07 | 33.83 |
| Baseline | Control | 144 | 32.18 | 8.85 | 30.72 | 33.64 |
| 3-Month | Experimental | 152 | 35.67 | 8.45 | 34.31 | 37.03 |
| 3-Month | Control | 141 | 32.24 | 8.92 | 30.75 | 33.73 |
| 6-Month | Experimental | 149 | 38.42 | 8.21 | 37.09 | 39.75 |
| 6-Month | Control | 138 | 32.31 | 9.05 | 30.79 | 33.83 |
| 9-Month | Experimental | 147 | 40.85 | 7.98 | 39.55 | 42.15 |
| 9-Month | Control | 136 | 32.45 | 9.12 | 30.90 | 34.00 |
| 12-Month | Experimental | 145 | 42.67 | 7.75 | 41.39 | 43.95 |
| 12-Month | Control | 134 | 32.52 | 9.18 | 30.95 | 34.09 |

**Table S7. Environmental Exposure Summary Statistics (Weekly Hours) - Experimental Group**

| **Time Period** | **n** | **Mean** | **SD** | **Median** | **Min** | **Max** | **IQR** |
| --- | --- | --- | --- | --- | --- | --- | --- |
| Month 1-3 | 152 | 3.85 | 2.42 | 3.50 | 0.5 | 10.2 | 2.8 |
| Month 4-6 | 149 | 4.72 | 2.78 | 4.25 | 0.8 | 11.5 | 3.2 |
| Month 7-9 | 147 | 5.18 | 2.95 | 4.75 | 1.0 | 12.0 | 3.5 |
| Month 10-12 | 145 | 5.42 | 3.12 | 5.00 | 1.2 | 12.4 | 3.8 |
| Overall | 145 | 4.79 | 2.82 | 4.38 | 0.9 | 11.5 | 3.3 |

**Table S8. Correlation Matrix for Study Variables at Baseline**

| **Variable** | **1** | **2** | **3** | **4** | **5** | **6** | **7** | **8** | **9** | **10** |
| --- | --- | --- | --- | --- | --- | --- | --- | --- | --- | --- |
| 1. Depression (PHQ-9) | 1.00 |  |  |  |  |  |  |  |  |  |
| 2. Anxiety (GAD-7) | 0.67** | 1.00 |  |  |  |  |  |  |  |  |
| 3. Attention Restoration | -0.51** | -0.44** | 1.00 |  |  |  |  |  |  |  |
| 4. Perceived Stress | 0.59** | 0.62** | -0.48** | 1.00 |  |  |  |  |  |  |
| 5. Nature Connectedness | -0.31** | -0.28** | 0.52** | -0.35** | 1.00 |  |  |  |  |  |
| 6. Cortisol | 0.42** | 0.45** | -0.38** | 0.51** | -0.25** | 1.00 |  |  |  |  |
| 7. HRV (RMSSD) | -0.35** | -0.38** | 0.41** | -0.44** | 0.29** | -0.47** | 1.00 |  |  |  |
| 8. Age | -0.18* | -0.15* | 0.19* | -0.22** | 0.14 | -0.12 | 0.08 | 1.00 |  |  |
| 9. Gender (Female=1) | 0.08 | 0.12* | 0.05 | 0.09 | 0.11 | 0.06 | -0.04 | -0.03 | 1.00 |  |
| 10. Education | -0.14* | -0.11 | 0.16* | -0.18* | 0.21** | -0.09 | 0.12 | 0.25** | 0.07 | 1.00 |

Note: *p < .05, **p < .01. N = 300.

**Table S9. Correlation Matrix for Study Variables at 12-Month Follow-up**

| **Variable** | **1** | **2** | **3** | **4** | **5** | **6** | **7** |
| --- | --- | --- | --- | --- | --- | --- | --- |
| 1. Depression (PHQ-9) | 1.00 |  |  |  |  |  |  |
| 2. Anxiety (GAD-7) | 0.71** | 1.00 |  |  |  |  |  |
| 3. Attention Restoration | -0.58** | -0.52** | 1.00 |  |  |  |  |
| 4. Perceived Stress | 0.64** | 0.68** | -0.55** | 1.00 |  |  |  |
| 5. Cortisol | 0.48** | 0.51** | -0.44** | 0.56** | 1.00 |  |  |
| 6. HRV (RMSSD) | -0.41** | -0.44** | 0.48** | -0.49** | -0.52** | 1.00 |  |
| 7. Environmental Exposure | -0.52** | -0.48** | 0.61** | -0.47** | -0.39** | 0.42** | 1.00 |

Note: *p < .05, **p < .01. N = 279.

**Table S10. Correlation Between Cumulative Environmental Exposure and Mental Health Outcomes**

| **Outcome Variable** | **Time Point** | **r** | **95% CI** | **p-value** |
| --- | --- | --- | --- | --- |
| Depression (PHQ-9) | 3-Month | -0.28 | [-0.38, -0.17] | < .001 |
| Depression (PHQ-9) | 6-Month | -0.38 | [-0.47, -0.28] | < .001 |
| Depression (PHQ-9) | 9-Month | -0.46 | [-0.54, -0.37] | < .001 |
| Depression (PHQ-9) | 12-Month | -0.52 | [-0.60, -0.43] | < .001 |
| Anxiety (GAD-7) | 3-Month | -0.24 | [-0.34, -0.13] | < .001 |
| Anxiety (GAD-7) | 6-Month | -0.35 | [-0.44, -0.25] | < .001 |
| Anxiety (GAD-7) | 9-Month | -0.42 | [-0.51, -0.32] | < .001 |
| Anxiety (GAD-7) | 12-Month | -0.48 | [-0.56, -0.39] | < .001 |

Note: Cumulative exposure calculated as total hours of biophilic art environment contact from baseline to each time point.

**Table S11. Repeated Measures ANOVA Summary for Depression Symptoms (PHQ-9)**

| **Source** | **Type III SS** | **df** | **Mean Square** | **F** | **p** | **Partial η²** | **Observed Power** |
| --- | --- | --- | --- | --- | --- | --- | --- |
| Between Subjects |  |  |  |  |  |  |  |
| Group | 847.32 | 1 | 847.32 | 47.32 | < .001 | .137 | .999 |
| Error (Between) | 5336.45 | 298 | 17.91 |  |  |  |  |
| Within Subjects |  |  |  |  |  |  |  |
| Time | 1458.72 | 4 | 364.68 | 23.85 | < .001 | .074 | .999 |
| Time × Group | 577.08 | 4 | 144.27 | 18.47 | < .001 | .058 | .999 |
| Error (Within) | 9126.84 | 1192 | 7.66 |  |  |  |  |

Note: Greenhouse-Geisser correction applied (ε = 0.89).

**Table S12. Repeated Measures ANOVA Summary for Anxiety Symptoms (GAD-7)**

| **Source** | **Type III SS** | **df** | **Mean Square** | **F** | **p** | **Partial η²** | **Observed Power** |
| --- | --- | --- | --- | --- | --- | --- | --- |
| Between Subjects |  |  |  |  |  |  |  |
| Group | 612.48 | 1 | 612.48 | 41.28 | < .001 | .122 | .999 |
| Error (Between) | 4421.67 | 298 | 14.84 |  |  |  |  |
| Within Subjects |  |  |  |  |  |  |  |
| Time | 1098.45 | 4 | 274.61 | 19.32 | < .001 | .061 | .999 |
| Time × Group | 510.24 | 4 | 127.56 | 17.15 | < .001 | .055 | .998 |
| Error (Within) | 8872.56 | 1192 | 7.44 |  |  |  |  |

Note: Greenhouse-Geisser correction applied (ε = 0.91).

**Table S13. Pairwise Comparisons for Depression Symptoms Between Groups at Each Time Point**

| **Time Point** | **Experimental Mean (SE)** | **Control Mean (SE)** | **Mean Difference** | **SE** | **95% CI** | **t** | **p (Bonferroni)** | **Cohen's d** |
| --- | --- | --- | --- | --- | --- | --- | --- | --- |
| Baseline | 8.82 (0.33) | 8.57 (0.35) | 0.25 | 0.48 | [-0.69, 1.19] | 0.52 | 1.000 | 0.06 |
| 3-Month | 7.45 (0.32) | 8.62 (0.36) | -1.17 | 0.48 | [-2.11, -0.23] | -2.44 | .023 | 0.29 |
| 6-Month | 6.12 (0.29) | 8.78 (0.37) | -2.66 | 0.47 | [-3.58, -1.74] | -5.66 | < .001 | 0.67 |
| 9-Month | 5.28 (0.26) | 8.91 (0.38) | -3.63 | 0.46 | [-4.53, -2.73] | -7.89 | < .001 | 0.94 |
| 12-Month | 4.67 (0.25) | 9.12 (0.39) | -4.45 | 0.46 | [-5.35, -3.55] | -9.67 | < .001 | 1.17 |

**Table S14. Pairwise Comparisons for Anxiety Symptoms Between Groups at Each Time Point**

| **Time Point** | **Experimental Mean (SE)** | **Control Mean (SE)** | **Mean Difference** | **SE** | **95% CI** | **t** | **p (Bonferroni)** | **Cohen's d** |
| --- | --- | --- | --- | --- | --- | --- | --- | --- |
| Baseline | 7.38 (0.30) | 7.21 (0.32) | 0.17 | 0.44 | [-0.69, 1.03] | 0.39 | 1.000 | 0.04 |
| 3-Month | 6.34 (0.28) | 7.35 (0.33) | -1.01 | 0.43 | [-1.86, -0.16] | -2.35 | .029 | 0.27 |
| 6-Month | 5.21 (0.26) | 7.52 (0.34) | -2.31 | 0.43 | [-3.15, -1.47] | -5.37 | < .001 | 0.64 |
| 9-Month | 4.45 (0.24) | 7.68 (0.35) | -3.23 | 0.42 | [-4.06, -2.40] | -7.69 | < .001 | 0.91 |
| 12-Month | 3.92 (0.22) | 7.85 (0.36) | -3.93 | 0.42 | [-4.76, -3.10] | -9.36 | < .001 | 1.12 |

**Table S15. Within-Group Change from Baseline at Each Time Point (Experimental Group)**

| **Outcome** | **Time Point** | **Mean Change** | **SE** | **95% CI** | **t** | **p** | **Cohen's d** |
| --- | --- | --- | --- | --- | --- | --- | --- |
| Depression | 3-Month | -1.37 | 0.28 | [-1.92, -0.82] | -4.89 | < .001 | 0.33 |
| Depression | 6-Month | -2.70 | 0.32 | [-3.33, -2.07] | -8.44 | < .001 | 0.65 |
| Depression | 9-Month | -3.54 | 0.35 | [-4.23, -2.85] | -10.11 | < .001 | 0.85 |
| Depression | 12-Month | -4.15 | 0.37 | [-4.88, -3.42] | -11.22 | < .001 | 1.00 |
| Anxiety | 3-Month | -1.04 | 0.25 | [-1.53, -0.55] | -4.16 | < .001 | 0.28 |
| Anxiety | 6-Month | -2.17 | 0.29 | [-2.74, -1.60] | -7.48 | < .001 | 0.58 |
| Anxiety | 9-Month | -2.93 | 0.31 | [-3.54, -2.32] | -9.45 | < .001 | 0.78 |
| Anxiety | 12-Month | -3.46 | 0.33 | [-4.11, -2.81] | -10.48 | < .001 | 0.92 |

**Table S16. Within-Group Change from Baseline at Each Time Point (Control Group)**

| **Outcome** | **Time Point** | **Mean Change** | **SE** | **95% CI** | **t** | **p** | **Cohen's d** |
| --- | --- | --- | --- | --- | --- | --- | --- |
| Depression | 3-Month | 0.05 | 0.31 | [-0.56, 0.66] | 0.16 | .873 | 0.01 |
| Depression | 6-Month | 0.21 | 0.34 | [-0.46, 0.88] | 0.62 | .537 | 0.05 |
| Depression | 9-Month | 0.34 | 0.36 | [-0.37, 1.05] | 0.94 | .348 | 0.08 |
| Depression | 12-Month | 0.55 | 0.38 | [-0.20, 1.30] | 1.45 | .149 | 0.12 |
| Anxiety | 3-Month | 0.14 | 0.28 | [-0.41, 0.69] | 0.50 | .618 | 0.04 |
| Anxiety | 6-Month | 0.31 | 0.31 | [-0.30, 0.92] | 1.00 | .319 | 0.08 |
| Anxiety | 9-Month | 0.47 | 0.33 | [-0.18, 1.12] | 1.42 | .158 | 0.12 |
| Anxiety | 12-Month | 0.64 | 0.35 | [-0.05, 1.33] | 1.83 | .069 | 0.16 |

**Table S17. Structural Equation Model Fit Indices**

| **Fit Index** | **Value** | **Acceptable Threshold** | **Interpretation** |
| --- | --- | --- | --- |
| Chi-square (χ²) | 187.45 | p > .05 | Model fit adequate |
| Degrees of freedom | 98 | — | — |
| χ²/df ratio | 1.91 | < 3.0 | Good fit |
| CFI | 0.96 | > 0.95 | Excellent fit |
| TLI | 0.95 | > 0.95 | Excellent fit |
| RMSEA | 0.048 | < 0.06 | Good fit |
| RMSEA 90% CI | [0.035, 0.061] | Upper CI < 0.08 | Good fit |
| SRMR | 0.052 | < 0.08 | Good fit |
| AIC | 12456.78 | Lower is better | — |
| BIC | 12678.34 | Lower is better | — |

**Table S18. Structural Equation Model Path Coefficients - Direct Effects**

| **Path** | **Unstandardized B** | **SE** | **Standardized β** | **95% CI** | **z** | **p** |
| --- | --- | --- | --- | --- | --- | --- |
| Environmental Exposure → Depression | -0.42 | 0.08 | -0.31 | [-0.47, -0.15] | -5.25 | < .001 |
| Environmental Exposure → Anxiety | -0.38 | 0.07 | -0.28 | [-0.42, -0.14] | -5.43 | < .001 |
| Environmental Exposure → Attention Restoration | 0.68 | 0.09 | 0.52 | [0.34, 0.70] | 7.56 | < .001 |
| Environmental Exposure → Stress Reduction | -0.54 | 0.08 | -0.45 | [-0.61, -0.29] | -6.75 | < .001 |
| Environmental Exposure → Environmental Perception | 0.62 | 0.08 | 0.48 | [0.32, 0.64] | 7.75 | < .001 |
| Attention Restoration → Depression | -0.48 | 0.07 | -0.38 | [-0.52, -0.24] | -6.86 | < .001 |
| Attention Restoration → Anxiety | -0.52 | 0.07 | -0.42 | [-0.56, -0.28] | -7.43 | < .001 |
| Stress Reduction → Depression | 0.35 | 0.06 | 0.32 | [0.20, 0.44] | 5.83 | < .001 |
| Stress Reduction → Anxiety | 0.42 | 0.07 | 0.38 | [0.24, 0.52] | 6.00 | < .001 |
| Environmental Perception → Depression | -0.28 | 0.06 | -0.22 | [-0.34, -0.10] | -4.67 | < .001 |
| Environmental Perception → Anxiety | -0.25 | 0.06 | -0.19 | [-0.31, -0.07] | -4.17 | < .001 |

**Table S19. Structural Equation Model Path Coefficients - Indirect Effects**

| **Indirect Path** | **Effect** | **Boot SE** | **95% CI (Bias-Corrected)** | **p** |
| --- | --- | --- | --- | --- |
| Exposure → Attention → Depression | -0.28 | 0.05 | [-0.38, -0.18] | < .001 |
| Exposure → Attention → Anxiety | -0.31 | 0.05 | [-0.41, -0.21] | < .001 |
| Exposure → Stress → Depression | -0.19 | 0.04 | [-0.27, -0.11] | < .001 |
| Exposure → Stress → Anxiety | -0.23 | 0.04 | [-0.31, -0.15] | < .001 |
| Exposure → Perception → Depression | -0.14 | 0.03 | [-0.20, -0.08] | < .001 |
| Exposure → Perception → Anxiety | -0.12 | 0.03 | [-0.18, -0.06] | < .001 |
| Total Indirect on Depression | -0.61 | 0.07 | [-0.75, -0.47] | < .001 |
| Total Indirect on Anxiety | -0.66 | 0.07 | [-0.80, -0.52] | < .001 |

Note: Bootstrap estimates based on 5,000 samples.

**Table S20. Mediation Analysis Summary - Proportion of Total Effect Mediated**

| **Mediator** | **Depression Outcome** | **Anxiety Outcome** |
| --- | --- | --- |
| Attention Restoration | 30.4% | 34.1% |
| Stress Reduction | 20.7% | 25.3% |
| Environmental Perception | 15.2% | 13.2% |
| Total Mediated | 66.3% | 72.6% |
| Direct Effect | 33.7% | 27.4% |

**Table S21. Moderation Analysis - Nature Connectedness × Group Interaction on Depression**

| **Variable** | **B** | **SE** | **β** | **t** | **p** | **95% CI** |
| --- | --- | --- | --- | --- | --- | --- |
| Intercept | 8.72 | 0.35 | — | 24.91 | < .001 | [8.03, 9.41] |
| Group (Experimental=1) | -2.85 | 0.48 | -0.34 | -5.94 | < .001 | [-3.79, -1.91] |
| Nature Connectedness | -0.18 | 0.04 | -0.19 | -4.50 | < .001 | [-0.26, -0.10] |
| Group × Nature Connectedness | -0.12 | 0.03 | -0.15 | -4.00 | < .001 | [-0.18, -0.06] |

Note: R² = .284, F(3, 275) = 36.42, p < .001. Outcome: 12-month PHQ-9 score.

**Table S22. Moderation Analysis - Nature Connectedness × Group Interaction on Anxiety**

| **Variable** | **B** | **SE** | **β** | **t** | **p** | **95% CI** |
| --- | --- | --- | --- | --- | --- | --- |
| Intercept | 7.28 | 0.32 | — | 22.75 | < .001 | [6.65, 7.91] |
| Group (Experimental=1) | -2.52 | 0.44 | -0.32 | -5.73 | < .001 | [-3.38, -1.66] |
| Nature Connectedness | -0.15 | 0.04 | -0.17 | -3.75 | < .001 | [-0.23, -0.07] |
| Group × Nature Connectedness | -0.09 | 0.03 | -0.12 | -3.00 | .003 | [-0.15, -0.03] |

Note: R² = .256, F(3, 275) = 31.58, p < .001. Outcome: 12-month GAD-7 score.

**Table S23. Moderation Analysis - Age × Group Interaction on Depression**

| **Variable** | **B** | **SE** | **β** | **t** | **p** | **95% CI** |
| --- | --- | --- | --- | --- | --- | --- |
| Intercept | 8.85 | 0.38 | — | 23.29 | < .001 | [8.10, 9.60] |
| Group (Experimental=1) | -2.42 | 0.52 | -0.29 | -4.65 | < .001 | [-3.44, -1.40] |
| Age | -0.04 | 0.02 | -0.12 | -2.00 | .047 | [-0.08, -0.00] |
| Group × Age | -0.05 | 0.02 | -0.11 | -2.50 | .013 | [-0.09, -0.01] |

Note: R² = .198, F(3, 275) = 22.67, p < .001. Outcome: 12-month PHQ-9 score.

**Table S24. Moderation Analysis - Age × Group Interaction on Anxiety**

| **Variable** | **B** | **SE** | **β** | **t** | **p** | **95% CI** |
| --- | --- | --- | --- | --- | --- | --- |
| Intercept | 7.42 | 0.35 | — | 21.20 | < .001 | [6.73, 8.11] |
| Group (Experimental=1) | -2.18 | 0.48 | -0.28 | -4.54 | < .001 | [-3.12, -1.24] |
| Age | -0.03 | 0.02 | -0.09 | -1.50 | .135 | [-0.07, 0.01] |
| Group × Age | -0.04 | 0.02 | -0.09 | -2.00 | .047 | [-0.08, -0.00] |

Note: R² = .175, F(3, 275) = 19.45, p < .001. Outcome: 12-month GAD-7 score.

**Table S25. Moderation Analysis - Baseline Symptom Severity × Group Interaction on Depression Change**

| **Variable** | **B** | **SE** | **β** | **t** | **p** | **95% CI** |
| --- | --- | --- | --- | --- | --- | --- |
| Intercept | -0.15 | 0.42 | — | -0.36 | .720 | [-0.98, 0.68] |
| Group (Experimental=1) | -3.28 | 0.58 | -0.39 | -5.66 | < .001 | [-4.42, -2.14] |
| Baseline PHQ-9 | 0.08 | 0.05 | 0.08 | 1.60 | .111 | [-0.02, 0.18] |
| Group × Baseline PHQ-9 | -0.15 | 0.04 | -0.18 | -3.75 | < .001 | [-0.23, -0.07] |

Note: R² = .312, F(3, 275) = 41.58, p < .001. Outcome: PHQ-9 change score (12-month minus baseline).

**Table S26. Clinical Response Rates by Group**

| **Response Category** | **Experimental Group n (%)** | **Control Group n (%)** | **χ²** | **p** | **OR** | **95% CI** |
| --- | --- | --- | --- | --- | --- | --- |
| Depression Response (≥50% reduction) | 98 (67.6%) | 24 (17.9%) | 71.24 | < .001 | 9.58 | [5.51, 16.66] |
| Depression Remission (PHQ-9 < 5) | 72 (49.7%) | 18 (13.4%) | 42.85 | < .001 | 6.38 | [3.52, 11.57] |
| Clinically Meaningful Depression Improvement (≥5 points) | 113 (77.9%) | 31 (23.1%) | 84.52 | < .001 | 11.67 | [6.73, 20.23] |
| Anxiety Response (≥50% reduction) | 94 (64.8%) | 21 (15.7%) | 69.82 | < .001 | 10.12 | [5.68, 18.03] |
| Anxiety Remission (GAD-7 < 5) | 85 (58.6%) | 22 (16.4%) | 53.18 | < .001 | 7.31 | [4.17, 12.81] |
| Clinically Meaningful Anxiety Improvement (≥5 points) | 107 (73.8%) | 26 (19.4%) | 82.74 | < .001 | 11.89 | [6.79, 20.82] |

Note: Based on completers at 12-month follow-up. Experimental n = 145, Control n = 134.

**Table S27. Dose-Response Analysis - Environmental Exposure Categories and Outcomes**

| **Exposure Category (Hours/Week)** | **n** | **Depression Change M (SD)** | **Anxiety Change M (SD)** | **Effect vs. Low Exposure (d)** |
| --- | --- | --- | --- | --- |
| Low (< 3 hours) | 38 | -1.82 (2.45) | -1.45 (2.28) | Reference |
| Moderate (3-5 hours) | 52 | -3.85 (2.78) | -3.21 (2.52) | 0.78 / 0.73 |
| High (5-8 hours) | 34 | -5.24 (2.92) | -4.38 (2.71) | 1.27 / 1.17 |
| Very High (> 8 hours) | 21 | -6.15 (3.05) | -5.12 (2.85) | 1.57 / 1.43 |

Note: Change scores represent 12-month minus baseline values. Negative values indicate symptom improvement.

**Table S28. Attrition Analysis - Comparison of Completers vs. Non-Completers**

| **Variable** | **Completers (n = 279) M (SD)** | **Non-Completers (n = 21) M (SD)** | **t / χ²** | **p** |
| --- | --- | --- | --- | --- |
| Age | 38.45 (12.42) | 34.86 (13.15) | 1.28 | .202 |
| Gender (% Female) | 56.6% | 61.9% | 0.24 | .625 |
| Education (% College+) | 60.9% | 52.4% | 0.58 | .446 |
| Baseline PHQ-9 | 8.62 (4.15) | 9.81 (4.58) | -1.31 | .192 |
| Baseline GAD-7 | 7.24 (3.75) | 8.05 (4.12) | -0.98 | .328 |
| Nature Connectedness | 18.72 (4.35) | 17.48 (4.82) | 1.28 | .201 |

Note: No significant differences between completers and non-completers on key variables.

**Table S29. Missing Data Patterns**

| **Variable** | **N Complete** | **N Missing** | **% Missing** | **Pattern** |
| --- | --- | --- | --- | --- |
| Baseline PHQ-9 | 300 | 0 | 0.0% | — |
| Baseline GAD-7 | 300 | 0 | 0.0% | — |
| 3-Month PHQ-9 | 293 | 7 | 2.3% | MCAR |
| 3-Month GAD-7 | 293 | 7 | 2.3% | MCAR |
| 6-Month PHQ-9 | 287 | 13 | 4.3% | MCAR |
| 6-Month GAD-7 | 287 | 13 | 4.3% | MCAR |
| 9-Month PHQ-9 | 283 | 17 | 5.7% | MCAR |
| 9-Month GAD-7 | 283 | 17 | 5.7% | MCAR |
| 12-Month PHQ-9 | 279 | 21 | 7.0% | MCAR |
| 12-Month GAD-7 | 279 | 21 | 7.0% | MCAR |
| GPS Exposure Data | 285 | 15 | 5.0% | MCAR |
| Cortisol Samples | 274 | 26 | 8.7% | MCAR |

Note: Little's MCAR test: χ²(45) = 52.34, p = .208, supporting missing completely at random assumption.

**Table S30. Sensitivity Analysis - Multiple Imputation Results**

| **Analysis** | **Depression Effect (B)** | **95% CI** | **Anxiety Effect (B)** | **95% CI** |
| --- | --- | --- | --- | --- |
| Complete Case | -4.45 | [-5.35, -3.55] | -3.93 | [-4.76, -3.10] |
| Multiple Imputation (m=20) | -4.38 | [-5.32, -3.44] | -3.87 | [-4.74, -3.00] |
| Last Observation Carried Forward | -4.22 | [-5.18, -3.26] | -3.78 | [-4.68, -2.88] |
| Mixed Model (ML estimation) | -4.41 | [-5.29, -3.53] | -3.91 | [-4.72, -3.10] |

Note: All estimates significant at p < .001. Results consistent across missing data handling approaches.

**Table S31. Biophilic Environment Assessment Scale (BEAS) Subscale Scores by Installation Site**

| **Subscale** | **Seoul Forest Park M (SD)** | **Jungnangcheon Stream M (SD)** | **Ttukseom Hangang Park M (SD)** | **F** | **p** |
| --- | --- | --- | --- | --- | --- |
| Natural Elements Presence | 24.8 (2.4) | 23.5 (2.8) | 25.2 (2.2) | 4.82 | .009 |
| Natural Materials Integration | 22.4 (3.1) | 24.6 (2.5) | 21.8 (3.3) | 8.45 | < .001 |
| Spatial Configuration | 23.7 (2.6) | 22.8 (3.0) | 24.5 (2.4) | 3.95 | .021 |
| Artistic Integration Quality | 25.1 (2.2) | 23.2 (2.9) | 24.8 (2.5) | 5.67 | .004 |
| Total BEAS Score | 96.0 (7.8) | 94.1 (8.5) | 96.3 (7.2) | 1.24 | .291 |

Note: Each subscale range: 6-30. Total scale range: 24-120. Ratings from trained environmental assessors (n = 4).

**Table S32. Reliability Analysis of Study Measures Across Time Points**

| **Measure** | **Baseline α** | **3-Month α** | **6-Month α** | **9-Month α** | **12-Month α** | **Average α** |
| --- | --- | --- | --- | --- | --- | --- |
| PHQ-9 | 0.89 | 0.90 | 0.88 | 0.91 | 0.89 | 0.89 |
| GAD-7 | 0.92 | 0.91 | 0.93 | 0.92 | 0.91 | 0.92 |
| Attention Restoration Scale | 0.90 | 0.89 | 0.91 | 0.90 | 0.92 | 0.90 |
| Perceived Stress Scale | 0.87 | 0.86 | 0.88 | 0.87 | 0.89 | 0.87 |
| Nature Relatedness Scale-6 | 0.85 | 0.86 | 0.84 | 0.87 | 0.86 | 0.86 |
| BEAS | 0.87 | — | 0.88 | — | 0.89 | 0.88 |

**Table S33. Variance Inflation Factors for Regression Models**

| **Predictor Variable** | **VIF** | **Tolerance** |
| --- | --- | --- |
| Group (Experimental vs. Control) | 1.12 | 0.893 |
| Age | 1.18 | 0.847 |
| Gender | 1.08 | 0.926 |
| Education Level | 1.24 | 0.806 |
| Baseline PHQ-9 | 2.15 | 0.465 |
| Baseline GAD-7 | 2.08 | 0.481 |
| Nature Connectedness | 1.45 | 0.690 |
| Environmental Exposure (Cumulative) | 1.38 | 0.725 |
| Attention Restoration | 1.72 | 0.581 |
| Perceived Stress | 1.85 | 0.541 |

Note: All VIF values < 3.0, indicating no problematic multicollinearity.

**Table S34. Hierarchical Linear Model Results for Depression Trajectory**

| **Fixed Effects** | **Estimate** | **SE** | **df** | **t** | **p** |
| --- | --- | --- | --- | --- | --- |
| Intercept | 8.68 | 0.32 | 298 | 27.13 | < .001 |
| Time (months) | -0.02 | 0.03 | 1113 | -0.67 | .505 |
| Group | -0.28 | 0.45 | 298 | -0.62 | .536 |
| Time × Group | -0.32 | 0.04 | 1113 | -8.00 | < .001 |

| **Random Effects** | **Variance** | **SD** |  |
| --- | --- | --- | --- |
| Intercept | 12.45 | 3.53 |  |
| Time Slope | 0.08 | 0.28 |  |
| Residual | 5.82 | 2.41 |  |

Note: ICC = 0.68. Deviance = 4528.34. AIC = 4542.34.

**Table S35. Hierarchical Linear Model Results for Anxiety Trajectory**

| **Fixed Effects** | **Estimate** | **SE** | **df** | **t** | **p** |
| --- | --- | --- | --- | --- | --- |
| Intercept | 7.28 | 0.29 | 298 | 25.10 | < .001 |
| Time (months) | 0.01 | 0.03 | 1113 | 0.33 | .739 |
| Group | -0.15 | 0.41 | 298 | -0.37 | .714 |
| Time × Group | -0.28 | 0.04 | 1113 | -7.00 | < .001 |

| **Random Effects** | **Variance** | **SD** |  |
| --- | --- | --- | --- |
| Intercept | 9.85 | 3.14 |  |
| Time Slope | 0.06 | 0.24 |  |
| Residual | 4.92 | 2.22 |  |

Note: ICC = 0.67. Deviance = 4312.56. AIC = 4326.56.

**Supplementary Code**

**R Code for Statistical Analysis**

# ============================================================

# Supplementary Analysis Code

# Long-term Effects of Urban Biophilic Art Environments

# ============================================================

# Load required packages

library(lme4)

library(lmerTest)

library(lavaan)

library(mediation)

library(ggplot2)

library(dplyr)

library(tidyr)

library(psych)

library(car)

library(emmeans)

library(mice)

# ============================================================

# 1. Data Preparation

# ============================================================

# Read data

data <- read.csv("biophilic_study_data.csv")

# Convert to long format for repeated measures

data_long <- data %>%

pivot_longer(

cols = c(PHQ9_T0, PHQ9_T1, PHQ9_T2, PHQ9_T3, PHQ9_T4,

GAD7_T0, GAD7_T1, GAD7_T2, GAD7_T3, GAD7_T4),

names_to = c(".value", "time"),

names_pattern = "(PHQ9|GAD7)_T(\\d)"

) %>%

mutate(

time = as.numeric(time),

time_months = time * 3,

group = factor(group, levels = c(0, 1),

labels = c("Control", "Experimental"))

)

# ============================================================

# 2. Descriptive Statistics

# ============================================================

# Summary statistics by group and time

descriptives <- data_long %>%

group_by(group, time_months) %>%

summarise(

n = n(),

PHQ9_mean = mean(PHQ9, na.rm = TRUE),

PHQ9_sd = sd(PHQ9, na.rm = TRUE),

PHQ9_se = PHQ9_sd / sqrt(n),

GAD7_mean = mean(GAD7, na.rm = TRUE),

GAD7_sd = sd(GAD7, na.rm = TRUE),

GAD7_se = GAD7_sd / sqrt(n),

.groups = "drop"

)

print(descriptives)

# ============================================================

# 3. Correlation Analysis

# ============================================================

# Baseline correlation matrix

baseline_vars <- data %>%

select(PHQ9_T0, GAD7_T0, attention_restoration_T0,

stress_T0, nature_connectedness, cortisol_T0,

hrv_T0, age, education)

cor_matrix <- cor(baseline_vars, use = "pairwise.complete.obs")

cor_test <- corr.test(baseline_vars, method = "pearson")

print(round(cor_matrix, 2))

print(cor_test$p)

# ============================================================

# 4. Repeated Measures ANOVA

# ============================================================

# Depression ANOVA

rm_anova_phq9 <- aov(PHQ9 ~ group * factor(time) +

Error(id/factor(time)), data = data_long)

summary(rm_anova_phq9)

# Anxiety ANOVA

rm_anova_gad7 <- aov(GAD7 ~ group * factor(time) +

Error(id/factor(time)), data = data_long)

summary(rm_anova_gad7)

# Effect sizes (partial eta squared)

library(effectsize)

eta_squared(rm_anova_phq9, partial = TRUE)

eta_squared(rm_anova_gad7, partial = TRUE)

# ============================================================

# 5. Hierarchical Linear Modeling

# ============================================================

# Depression trajectory model

hlm_phq9 <- lmer(PHQ9 ~ time_months * group + (1 + time_months | id),

data = data_long, REML = FALSE)

summary(hlm_phq9)

confint(hlm_phq9)

# Anxiety trajectory model

hlm_gad7 <- lmer(GAD7 ~ time_months * group + (1 + time_months | id),

data = data_long, REML = FALSE)

summary(hlm_gad7)

confint(hlm_gad7)

# ICC calculation

library(performance)

icc(hlm_phq9)

icc(hlm_gad7)

# ============================================================

# 6. Structural Equation Modeling

# ============================================================

# Define SEM model

sem_model <- '

# Measurement model (if using latent variables)

# Structural model - Direct effects

PHQ9_T4 ~ c1*exposure_cumulative + c2*attention_T4 + c3*stress_T4 + c4*perception_T4

GAD7_T4 ~ d1*exposure_cumulative + d2*attention_T4 + d3*stress_T4 + d4*perception_T4

# Mediator regressions

attention_T4 ~ a1*exposure_cumulative

stress_T4 ~ a2*exposure_cumulative

perception_T4 ~ a3*exposure_cumulative

# Indirect effects

ind_att_phq := a1*c2

ind_str_phq := a2*c3

ind_per_phq := a3*c4

ind_att_gad := a1*d2

ind_str_gad := a2*d3

ind_per_gad := a3*d4

# Total indirect effects

total_ind_phq := ind_att_phq + ind_str_phq + ind_per_phq

total_ind_gad := ind_att_gad + ind_str_gad + ind_per_gad

# Total effects

total_phq := c1 + total_ind_phq

total_gad := d1 + total_ind_gad

'

# Fit SEM model

sem_fit <- sem(sem_model, data = data, se = "bootstrap", bootstrap = 5000)

summary(sem_fit, fit.measures = TRUE, standardized = TRUE, ci = TRUE)

# Model fit indices

fitMeasures(sem_fit, c("chisq", "df", "pvalue", "cfi", "tli",

"rmsea", "rmsea.ci.lower", "rmsea.ci.upper", "srmr"))

# ============================================================

# 7. Moderation Analysis

# ============================================================

# Nature connectedness moderation on depression

mod_nc_phq9 <- lm(PHQ9_T4 ~ group * nature_connectedness + PHQ9_T0,

data = data)

summary(mod_nc_phq9)

# Simple slopes analysis

library(interactions)

sim_slopes(mod_nc_phq9, pred = group, modx = nature_connectedness)

interact_plot(mod_nc_phq9, pred = group, modx = nature_connectedness)

# Age moderation

mod_age_phq9 <- lm(PHQ9_T4 ~ group * age + PHQ9_T0, data = data)

summary(mod_age_phq9)

# Baseline severity moderation

mod_base_phq9 <- lm(PHQ9_change ~ group * PHQ9_T0, data = data)

summary(mod_base_phq9)

# ============================================================

# 8. Missing Data Analysis

# ============================================================

# Little's MCAR test

library(naniar)

mcar_test(data_long)

# Multiple imputation

imp <- mice(data, m = 20, method = "pmm", seed = 123)

# Analysis with imputed data

fit_imp <- with(imp, lm(PHQ9_T4 ~ group + PHQ9_T0 + age + gender))

pooled <- pool(fit_imp)

summary(pooled)

# ============================================================

# 9. Sensitivity Analysis

# ============================================================

# Complete case analysis

complete_data <- data_long %>% filter(complete.cases(.))

sens_complete <- lmer(PHQ9 ~ time_months * group + (1 | id),

data = complete_data)

# LOCF analysis

data_locf <- data_long %>%

group_by(id) %>%

fill(PHQ9, GAD7, .direction = "down") %>%

ungroup()

sens_locf <- lmer(PHQ9 ~ time_months * group + (1 | id), data = data_locf)

# Compare results

compare_sensitivity <- data.frame(

Method = c("Complete Case", "LOCF", "Mixed Model"),

Estimate = c(fixef(sens_complete)["time_months:groupExperimental"],

fixef(sens_locf)["time_months:groupExperimental"],

fixef(hlm_phq9)["time_months:groupExperimental"])

)

print(compare_sensitivity)

# ============================================================

# 10. Effect Size Calculations

# ============================================================

# Cohen's d at each time point

effect_sizes <- data_long %>%

group_by(time_months) %>%

summarise(

d_phq9 = (mean(PHQ9[group == "Control"], na.rm = TRUE) -

mean(PHQ9[group == "Experimental"], na.rm = TRUE)) /

sqrt((var(PHQ9[group == "Control"], na.rm = TRUE) +

var(PHQ9[group == "Experimental"], na.rm = TRUE)) / 2),

d_gad7 = (mean(GAD7[group == "Control"], na.rm = TRUE) -

mean(GAD7[group == "Experimental"], na.rm = TRUE)) /

sqrt((var(GAD7[group == "Control"], na.rm = TRUE) +

var(GAD7[group == "Experimental"], na.rm = TRUE)) / 2),

.groups = "drop"

)

print(effect_sizes)

# ============================================================

# 11. Visualization

# ============================================================

# Trajectory plot for depression

p1 <- ggplot(descriptives, aes(x = time_months, y = PHQ9_mean,

color = group, linetype = group)) +

geom_line(size = 1.2) +

geom_point(size = 3) +

geom_errorbar(aes(ymin = PHQ9_mean - 1.96*PHQ9_se,

ymax = PHQ9_mean + 1.96*PHQ9_se),

width = 0.5) +

scale_x_continuous(breaks = c(0, 3, 6, 9, 12)) +

labs(x = "Time (Months)", y = "PHQ-9 Score",

title = "Depression Symptom Trajectories",

color = "Group", linetype = "Group") +

theme_minimal() +

theme(legend.position = "bottom")

ggsave("depression_trajectory.png", p1, width = 8, height = 6, dpi = 300)

# Trajectory plot for anxiety

p2 <- ggplot(descriptives, aes(x = time_months, y = GAD7_mean,

color = group, linetype = group)) +

geom_line(size = 1.2) +

geom_point(size = 3) +

geom_errorbar(aes(ymin = GAD7_mean - 1.96*GAD7_se,

ymax = GAD7_mean + 1.96*GAD7_se),

width = 0.5) +

scale_x_continuous(breaks = c(0, 3, 6, 9, 12)) +

labs(x = "Time (Months)", y = "GAD-7 Score",

title = "Anxiety Symptom Trajectories",

color = "Group", linetype = "Group") +

theme_minimal() +

theme(legend.position = "bottom")

ggsave("anxiety_trajectory.png", p2, width = 8, height = 6, dpi = 300)

# ============================================================

# 12. Power Analysis

# ============================================================

library(pwr)

# Post-hoc power analysis

pwr.t.test(n = 145, d = 0.84, sig.level = 0.05, type = "two.sample")

# Achieved power for ANOVA

pwr.f2.test(u = 4, v = 1192, f2 = 0.058/(1-0.058), sig.level = 0.05)

# ============================================================

# End of Analysis Code

# ============================================================

**SPSS Syntax for Key Analyses**

* ============================================================

* SPSS Syntax for Supplementary Analyses

* Long-term Effects of Urban Biophilic Art Environments

* ============================================================.

* Descriptive Statistics.

DESCRIPTIVES VARIABLES=PHQ9_T0 PHQ9_T1 PHQ9_T2 PHQ9_T3 PHQ9_T4

GAD7_T0 GAD7_T1 GAD7_T2 GAD7_T3 GAD7_T4

/STATISTICS=MEAN STDDEV MIN MAX SKEWNESS KURTOSIS.

* Split by group.

SORT CASES BY group.

SPLIT FILE BY group.

DESCRIPTIVES VARIABLES=PHQ9_T0 PHQ9_T1 PHQ9_T2 PHQ9_T3 PHQ9_T4

/STATISTICS=MEAN STDDEV.

SPLIT FILE OFF.

* Correlation Matrix.

CORRELATIONS

/VARIABLES=PHQ9_T0 GAD7_T0 attention_T0 stress_T0

nature_connectedness cortisol_T0 hrv_T0 age

/PRINT=TWOTAIL NOSIG

/STATISTICS DESCRIPTIVES

/MISSING=PAIRWISE.

* Repeated Measures ANOVA for Depression.

GLM PHQ9_T0 PHQ9_T1 PHQ9_T2 PHQ9_T3 PHQ9_T4 BY group

/WSFACTOR=time 5 Polynomial

/METHOD=SSTYPE(3)

/PLOT=PROFILE(time*group) TYPE=LINE ERRORBAR=CI MEANREFERENCE=NO

/EMMEANS=TABLES(group*time) COMPARE(group) ADJ(BONFERRONI)

/PRINT=DESCRIPTIVE ETASQ OPOWER HOMOGENEITY

/CRITERIA=ALPHA(.05)

/WSDESIGN=time

/DESIGN=group.

* Repeated Measures ANOVA for Anxiety.

GLM GAD7_T0 GAD7_T1 GAD7_T2 GAD7_T3 GAD7_T4 BY group

/WSFACTOR=time 5 Polynomial

/METHOD=SSTYPE(3)

/PLOT=PROFILE(time*group) TYPE=LINE ERRORBAR=CI MEANREFERENCE=NO

/EMMEANS=TABLES(group*time) COMPARE(group) ADJ(BONFERRONI)

/PRINT=DESCRIPTIVE ETASQ OPOWER HOMOGENEITY

/CRITERIA=ALPHA(.05)

/WSDESIGN=time

/DESIGN=group.

* Moderation Analysis - Nature Connectedness.

PROCESS y=PHQ9_T4/x=group/w=nature_connectedness/cov=PHQ9_T0/model=1

/jn=1/plot=1/meanc=1.

* Moderation Analysis - Age.

PROCESS y=PHQ9_T4/x=group/w=age/cov=PHQ9_T0/model=1

/jn=1/plot=1/meanc=1.

* Mediation Analysis.

PROCESS y=PHQ9_T4/x=exposure_cumulative/m=attention_T4 stress_T4 perception_T4

/cov=PHQ9_T0/model=4/boot=5000/seed=12345.

* Reliability Analysis.

RELIABILITY

/VARIABLES=PHQ9_item1 PHQ9_item2 PHQ9_item3 PHQ9_item4 PHQ9_item5

PHQ9_item6 PHQ9_item7 PHQ9_item8 PHQ9_item9

/SCALE('PHQ-9') ALL

/MODEL=ALPHA

/STATISTICS=DESCRIPTIVE SCALE CORR.

RELIABILITY

/VARIABLES=GAD7_item1 GAD7_item2 GAD7_item3 GAD7_item4

GAD7_item5 GAD7_item6 GAD7_item7

/SCALE('GAD-7') ALL

/MODEL=ALPHA

/STATISTICS=DESCRIPTIVE SCALE CORR.

* Multicollinearity Diagnostics.

REGRESSION

/MISSING LISTWISE

/STATISTICS COEFF OUTS CI(95) R ANOVA COLLIN TOL

/CRITERIA=PIN(.05) POUT(.10)

/NOORIGIN

/DEPENDENT PHQ9_T4

/METHOD=ENTER group age gender education PHQ9_T0

nature_connectedness exposure_cumulative.

* Effect Size Calculation.

COMPUTE d_PHQ9_T4 = (MEAN(PHQ9_T4 IF group=0) - MEAN(PHQ9_T4 IF group=1)) /

SQRT((VARIANCE(PHQ9_T4 IF group=0) + VARIANCE(PHQ9_T4 IF group=1)) / 2).

EXECUTE.

* Clinical Response Rates.

COMPUTE PHQ9_response = (PHQ9_T0 - PHQ9_T4) / PHQ9_T0 >= 0.5.

COMPUTE PHQ9_remission = PHQ9_T4 < 5.

COMPUTE PHQ9_meaningful = (PHQ9_T0 - PHQ9_T4) >= 5.

EXECUTE.

CROSSTABS

/TABLES=PHQ9_response PHQ9_remission PHQ9_meaningful BY group

/FORMAT=AVALUE TABLES

/STATISTICS=CHISQ PHI RISK

/CELLS=COUNT ROW COLUMN TOTAL.

* Missing Data Analysis - Little's MCAR Test.

MVA VARIABLES=PHQ9_T0 PHQ9_T1 PHQ9_T2 PHQ9_T3 PHQ9_T4

GAD7_T0 GAD7_T1 GAD7_T2 GAD7_T3 GAD7_T4

/MPATTERN

/TTEST

/MCAR.

* ============================================================

* End of SPSS Syntax

* ============================================================.

**End of Supplementary File 1**
